# Analytical Rheology of Honey: A State-of-the-Art Review

**DOI:** 10.3390/foods10081709

**Published:** 2021-07-23

**Authors:** Célia Faustino, Lídia Pinheiro

**Affiliations:** iMed.Ulisboa—Research Institute for Medicines, Faculty of Pharmacy, Universidade de Lisboa, Av. Prof. Gama Pinto, 1649-003 Lisboa, Portugal; cfaustino@ff.ulisboa.pt

**Keywords:** honey, viscosity, rheology, moisture, temperature, adulteration

## Abstract

Honey has been used as a nutraceutical product since ancient times due to its nutritional and medicinal properties. Honey rheology influences its organoleptic properties and is relevant for processing and quality control. This review summarizes the rheological behaviour of honeys of different botanical source(s) and geographical locations that has been described in the literature, focusing on the relation between rheological parameters, honey composition (moisture, water activity, sugar content, presence of colloidal matter) and experimental conditions (temperature, time, stress, shear rate). Both liquid and crystallized honeys have been addressed. Firstly, the main mathematical models used to describe honey rheological behaviour are presented highlighting moisture and temperature effects. Then, rheological data from the literature regarding distinct honey types from different countries is analysed and results are compared. Although most honeys are Newtonian fluids, interesting shear-thinning and thixotropic as well as anti-thixotropic behaviour have been described for some types of honey. Rheological parameters have also been successfully applied to identify honey adulteration and to discriminate between different honey types. Several chemometric techniques have also been employed to obtain the complex relationships between honey physicochemical and rheological properties, including partial least squares (PLS), principal component analysis (PCA) and artificial neural networks (ANN).

## 1. Introduction

Honey is a complex food matrix produced by honeybees (*Apis mellifera*) from the nectar collected from plants (Blossom or nectar honey), or from the excretions of plant-sucking insects on the living parts of plants or secretions of living parts of plants (honeydew honey), which the bees transform and mix with their own specific substances [1]. Honey is mainly a supersaturated solution of sugars (mostly fructose, glucose and some sucrose) with low water content and small concentrations of bioactive compounds, such as phenolic acids, flavonoids and other polyphenolic compounds, carotenoids, organic acids, amino acids, peptides, proteins, enzymes, lipids, waxes, aroma compounds, vitamins, minerals and pollen grains [1,2,3].

The large variation in the chemical composition of honeys, which depends on floral sources, climate, harvesting process, storage conditions and ageing, contributes to the huge diversity of colour, aroma, flavour and viscosity of honeys [2,4]. The high osmolarity of honey, due to its high sugar content, combined with its low water activity (below 0.60) are usually associated with high viscosity. Along with the acidic pH of honey (pH 3.2–4.5), these properties have been linked to non-specific honey’s antimicrobial activity [2,5,6].

The food industry has been challenged by constant technological innovation, where the transfer of methodologies and technologies from other industrial sectors has served these purposes. In this context, rheology, as the science studying the flow and deformation of materials under well-defined conditions, has come to assume a significant role in the food industry [2,7,8,9,10,11,12]. Consistency and other mechanical characteristics in the rheology domain influence the industrial processing of foods (usually comprising mixing/stirring, pumping, dosing, dispersing, extrusion, spinning, coating, injection moulding and spraying), product texture and stability, and the preparation and consumption of food. The impact of rheology is also manifested in oral perception and digestion, resulting from the effects on flow characteristics of these mechanical and chemical processes [7,9,10]. The structural breakdown during food mastication is associated with textural sensation, and rheological measurements may, thus, be employed to control other quality attributes such as flavour or nutrient release at specific sites [7,9,10].

Adequate knowledge of the rheological properties of foods has been shown to be relevant [7,9,10,12,13,14,15,16] in (i) the design of processing lines (where the flow type, determined by the food rheological behaviour, influences parameters such as pipes, pumps and tanks sizing); (ii) in quality control (of raw materials, intermediate and final product); (iii) in process control (for example, assuring a more efficacious control of the product characteristics, measuring viscosity through on-line installation); (iv) in the characterization of the structure and the functionality of foods (as a way to understand the structure of the food or the distribution of its molecular components, especially of macromolecular components, as well as predicting structural variations during preparation processes, packaging and storage strategies); and (v) in product development (when, for example, texture and consistency analysis enables progress in food development without resorting to a sensory panel, and prior to the valuable relationships between sensory and rheological assessment indispensable for consumer acceptance).

Honey’s rheological properties are relevant to consumers, honey keepers, processors and handlers since rheological parameters provide useful information that allows the development of new products, optimization of industrial processes and control of the quality and authenticity of honeys. This review of the current literature on honey rheology highlights the contribution of rheological parameters to the intrinsic properties of honey, as well as the influence of time and temperature on honey behaviour.

## 2. Rheological Models

Rheological classification encompasses the quantification of the functional relationships between stress, deformation, and the consequential rheological features such as elasticity, viscosity, or viscoelasticity [7,9,10].

Rheological measurements are generally performed either by applying a small force (stress) and measuring the deformation of the sample (strain), or a settled amount of movement (strain) and measuring the stress developed in the sample [7,17].

The correlation of shear stress and shear strain can be employed to depict the rheological data of food systems through several flow models. Functional models may also be established accounting for the influence of state variables (such as temperature) and foods structure/composition [17].

Most honeys are Newtonian fluids [18,19,20,21,22,23,24,25,26] characterized by constant viscosity (*η*) at a fixed temperature, which can be described by Newton’s law for flow (Equation (1)) showing a linear relation between shear stress (*σ*) and shear rate ($\dot{\gamma }$):

(1) $$\;\sigma =\eta \dot{\gamma }$$

However, some honeys, including heather honey, New Zealand manuka honey, Indian karvi honey, Nigerian honey, buckwheat, white-clover honey and several eucalyptus honeys show non-Newtonian behaviour, with viscosity values that change with shear rate at constant temperature [2,8,11,23,27,28,29,30,31,32,33,34,35,36,37,38,39,40,41,42,43,44,45]. For non-Newtonian fluids, the shear stress (SS)/shear rate (SR) ratio defines the apparent viscosity (*η*_app_) of the fluid at constant temperature, similarly to that of Newtonian fluids; however, this coefficient changes with SR while the dynamic viscosity of Newtonian fluids is SR-independent. Moreover, a thixotropic effect has also been observed, corresponding to a decrease in viscosity with time at constant shear rate and temperature [12,14,29,30]. The non-Newtonian behaviour may be related to the existence of colloidal matter, such as high molecular weight sugars and proteins, which accounts also for the usually observed thixotropic property [23,31,32,33,46,47].

**Figure 1:**
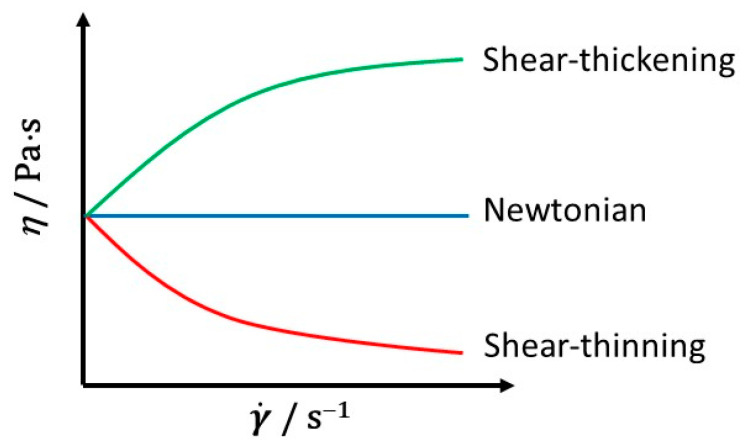
Common types of rheological behaviour. Honey samples usually show Newtonian or shear-thinning flow curves.

Non-Newtonian behaviour of honey has been described in the literature mostly by using the Ostwald-de Waele (also known as Power-law) model (Equation (2)):

(2) $$\;\sigma =K{\dot{\gamma }}^{n}$$

where *K* is the consistency factor and *n* is the flow behaviour index. Shear thinning fluids whose viscosity decreases with increasing shear rate have 0 < *n* < 1, while shear thickening fluids, characterized by increasing viscosity with shear rate, have 1 < *n* < ∞. For Newtonian fluids, *n* = 1 and *K* = *η* (Figure 1). In the limit of very low and very high shear rates the viscosity of non-Newtonian fluids is constant, and the two limiting values are known as zero-shear (*η*_0_) and infinite-shear (*η*_∞_) viscosities, respectively [2,48]. Nevertheless, the Power-law model does not portrait the low-shear and high-shear rate constant viscosity data of shear-thinning honeys and other food samples [17].

In structured and concentrated food materials an apparent solid-to-liquid flow transition (referred as yielding) can occur, depending on the material structure and the applied shear stress. Thus, the yield stress is the minimum shear stress required to initiate a flow transition [10]. Despite its importance in practical terms, it is however a questionable theoretical issue for many researchers, since below its value elastic behaviour of a material is always followed by some flow; however, when compared to the material elasticity, this flow is too small and can be omitted [17,33]. Since the yielding issue is an engineering reality in many food products, the inclusion of the yield stress in the Power-law leads to the Herschel–Bulkley model (Equation (3)),

(3) $$\;\sigma =\sigma_{0}+K{\dot{\gamma }}^{n}$$

where *σ*_0_ is the yield stress.

On the other hand, numerous food dispersions are rheologically characterized by the Bingham plastic model (Equation (4)) and the Casson model (Equation (5)), which have been occasionally employed to interpret honey flow behaviour [7,11,15,17,24,31,38,43,46,49,50,51].

(4) $$\;\sigma =\sigma_{0}+K\dot{\gamma }$$

(5) $$\;\sigma^{0.5}=\sigma_{0}{}^{0.5}+K{\dot{\gamma }}^{0.5}$$

Time-dependency rheological behaviour of honey has been characterized by the Weltman model (Equation (6)),

(6)  σ=A−B(lnt)

where *A* is the shear stress at *t* = 1 s (Pa), *B* is a time coefficient of thixotropic breakdown, and *t* is the shearing time (s) [17,32,33]; *B* takes negative or positive values, respectively, in thixotropic and anti-thixotropic behaviour [17,33].

Dynamic measurements have also been performed in many honeys. The mechanical response appears in the form of a strain (or stress) and is recorded as a function of time. The time scale probed is resolved by the angular frequency of oscillation, *ω*, of the shear deformation. When the stress is proportional to the strain deformation, the food material can be designated as an ideal elastic solid with the proportional constant being called the shear modulus of the material; in this case, the stress is in phase with the imposed sinusoidal strain deformation. On the other hand, an ideal viscous material responds with a π/2 out-of-phase, and the sample stress is proportional to the rate of deformation, with the fluid viscosity being the proportionality constant [7,9,19,24,38,52,53]. Viscoelastic food materials will thus comprise both in-phase and out-of-phase contributions being characterised by the storage (elastic) modulus, *G*′(*ω*), and the loss (viscous) modulus, *G*′(*ω*), following the stress response of the viscoelastic material *σ*(*t*) to a sinusoidal strain deformation *γ*(*t*)

(7) $$\;\sigma \left(t\right)=G\prime \left(\omega \right)\gamma_{0}\sin\left(\omega t\right)+G\prime \left(\omega \right)\gamma_{0}\cos\left(\omega t\right)$$

The overall response of the food material against the sinusoidal strain can be characterized by using the complex modulus G* (Equation (8)) and the complex viscosity η* (Equation (9)), respectively:

(8) $$\;G^{*}=\sqrt{{\left(G\prime \right)}^{2}+{\left(G\prime \right)}^{2}}$$

(9) $$\eta^{*}=G^{*}/\omega$$

The storage modulus, *G*′, and the loss modulus, *G*′, describe the energy storage and the energy dissipation in the flow, respectively. Both moduli are commonly modelled as a Power function of oscillatory frequency (Equations (10) and (11)) in the description of viscoelastic behaviour of food and dispersions [25,29,33]:

(10) $$\;G^{\prime }=K^{\prime }\omega^{n^{\prime }}$$

(11) $$G^{\prime \prime }=K^{\prime \prime }\omega^{n^{\prime \prime }}$$

The intercepts *K*′ and *K*′ and the slopes *n*′ and *n*′ are, respectively, the consistency coefficient and the behaviour index in Power-law viscoelastic properties [25,29,33].

Another material function frequently used to describe the viscoelastic behaviour is the tangent of the phase shift (or phase angle), a ratio between viscous and elastic properties, which is given by (Equation (12)):

(12) $$\tan\delta \left(\omega \right)=\frac{G\prime \prime \left(\omega \right)}{G\prime \left(\omega \right)}$$

where *δ* represents the phase difference between the applied strain and the response stress. For an entirely elastic material (Hookean body) *δ* is equal to 0°, while for Newtonian fluids the value is 90° [7,9,15,19]. Values of tan*δ* < 1 imply a high particle association due to colloidal forces [21]. Viscoelastic behaviour can be observed in food materials exhibiting both viscous and elastic properties [7,9,19]. Honey is a complex viscoelastic material characterized by liquid-like rheological behaviour, where the magnitude of *G*′′ is usually much higher than that of *G*′ [15,19,38,49].

In food systems, one can observe the correlation between steady shear and dynamic shear parameters (at equivalent angular frequency and shear rate values), and steady shear viscosity can be predicted from complex shear viscosity (and vice versa) using the empirical Cox-Merz rule [7,9,19,24,39,54]. The Cox-Merz rule is thus defined by the correspondence between the shear-rate dependence of the steady shear viscosity, and the frequency dependence of the complex viscosity:

(13) $${\left|\eta^{*}\left(\omega \right)=\eta (\dot{\gamma })\right|}_{\omega =\dot{\gamma }}$$

A modified Cox-Merz equation can also be used (Equation (14)):

(14) $${\left|\alpha \cdot \eta^{*}\left(\omega \right)=\eta (\dot{\gamma })\right|}_{\omega =\dot{\gamma }}$$

where *α* is a shift factor that can superimpose the *η*^∗^ on *η*.

Deviations from the Cox-Merx rule may be an indication of structural heterogeneities in a food [9]. This relationship has been observed in several honeys from different botanical and geographical origin [11,24,38,39,41]. However, it has not been possible to apply it to honeys in the crystallized state [7,17], nor to creamed honey or heather honeys which form a dense gel-like fluid structure [32,39].

In the cases where the Cox-Mertz rule is not observed, a Power-law function (Equation (15)) is fitted to the steady and dynamic viscosity,

(15) $${\left|\eta^{*}\left(\omega \right)=K{\left[\eta (\dot{\gamma })\right]}^{\beta }\right|}_{\omega =\dot{\gamma }}$$

where *Κ* and *β* are constants of the model, determined by nonlinear regression of the experimental data.

Viscoelastic food materials are also studied through transient tests (providing creep and creep-recovery curves) which describe the time dependence of viscoelasticity allowing to establish their texture stability. Applying and removing an instantaneous shear stress for a pre-established time period, creep and recovery phases can depict a possible structural collapse of food materials. Massless mechanical models such as those of Maxwell, Kelvin-Voigt or Burgers can be used for the interpretation of results [7,9,51,55,56]. These mechanical models are comprised of springs and dashpots that can be arranged either in series (Maxwell model) or in parallel (Kelvin-Voigt model). The spring is considered an ideal solid Hookean element, and the dashpot is regarded as an ideal fluid Newtonian element. The stress-strain relations in dashpot and spring can be expressed, respectively, as in Equation (1) and in Equation (16):

(16) G=σ/γ

where *G* is the elastic modulus of a solid and *γ* represent the strain.

In the Maxwell model, both elements (spring and dashpot), although subjected to the same stress, are allowed an independent strain. The total strain rate is the sum of the elastic and the viscous contributions, so that

(17) $$\dot{\gamma }\left(t\right)=\frac{\sigma }{\eta }+\frac{1}{G}\;\frac{d\sigma }{dt}$$

Although it does not consider the equilibrium stress, the Maxwell model can be useful in understanding stress relaxation data. In stress relaxation testing, the stress required to maintain the deformation caused by a constant strain applied is measured as a function of time [7,55].

In creep experiments, a constant stress is applied to the food material and the corresponding strain is measured as a function of time. The related parameters are the creep compliance *J* (the ratio of strain to stress) and the relaxation time *λ*_rel_ (the ratio of viscosity to elastic modulus). When subjected to a sudden force, the response of a material can be detected using the Kelvin-Voigt model, which represents the start point for the development of mechanical analogues describing the creep behaviour.

In the Kelvin-Voigt model, also known as the Voigt model, spring and dashpot are subjected to the same strain but different stresses. The total stress is the sum of the stress in each element, such that the corresponding stress–strain relation can be written as (Equation (18)):

(18) $$\sigma =G\gamma +\dot{\eta \gamma \;}$$

The Kelvin-Voigt configuration is not broad enough to model creep in many biological materials [7,55]. This drawback can be overcome using the Burgers model, which is a Maxwell and a Kelvin model associated in series [7,51,55,56]. The Burgers model (Equation (19)), which may describe the complete creep and recovery curve in creep experiments and is widely used in food systems (for example proteins, polysaccharides, and their composite gels), can be expressed in terms of the system deformation per unit stress, namely compliance (*J*):

(19) $$J\left(t\right)=\frac{1}{G_{0}}+\frac{1}{G_{1}}\;\left[1-\exp\left(\frac{-tG_{1}}{{}_{1}}\right)\right]+\frac{t}{\eta_{0}}\;$$

where *J*(*t*) is the overall compliance at any time in the creep phase, *G*_0_ is the instantaneous elastic modulus of the Maxwell unit (indicating the gel rigidity or strength), *η*_0_ is the viscosity of the liquid filling the dashpot of the Maxwell element (Pa s), *G*_1_ is the shear modulus of the Kelvin-Voigt unit (illustrating the gel-cohesive force and the resistance to deformation caused by the three-dimensional network), and *η*_1_ is the viscosity of the liquid filling the dashpot of the Kelvin-Voigt element (Pa s). The values of *G*_0_, *G*_1_, *η*_0_ and *η*_1_ can be employed to understand the internal structure of a food product [51,56].

## 3. Rheological Dependence on Temperature and Honey Composition

The rheological parameters of honey are influenced by several factors such as the composition and temperature. Increasing temperature leads to a decrease of the average intermolecular forces and viscosity. Thus, the viscosity of honey usually decreases with increasing temperature due to less molecular friction and reduced hydrodynamic forces [8,23,41,57,58].

The most useful model to evaluate the temperature–honey–viscosity relationship is not a consensual issue in the literature; naturally, the choice will depend on the temperature range under study, the physicochemical phenomena involved, and the predictive capacity of the model [39].

The Arrhenius model (Equation (20)) has been that most often used to adequately describe the dependence of viscosity on temperature [8,18,20,23,31,38,39,40,41,58,59,60]

(20) $$\eta =\eta_{0}\;\exp\left(\frac{E_{a}}{RT}\right)$$

where *E*_a_ is the activation energy (kJ mol^−1^) reflecting the sensitivity of viscosity to temperature variations [20,33,38,39,40,41,58,59], and the pre-exponential factor (*η*_0_) represents viscosity at a temperature close to infinity.

However, this model generates a relatively high value of *E*_a_ [41]. Other models, such as the William-Landel-Ferry (WLF), the Vogel-Taumann-Fulcher (VTF) and the Power-law, have been employed with success [8,18,19,23,24,35,38,39,41,58].

The William-Landel-Ferry (WLF) model uses glass-transition temperature (*T*_g_) and viscosity in the glass state (*η*_g_) to describe the dynamic viscosity of honey [8,18,19,23,24,35,39,58]. The model allows for identification of glass-transition temperature based on rheological measurements and is frequently applied in polymer systems (Equation (21)):

(21) $$\ln\left(\frac{\eta }{\eta_{g}}\right)=\frac{-C_{1}\left(T-T_{g}\right)}{C_{2}+\left(T-T_{g}\right)}\;$$

where *η*_g_ is the viscosity at *T*_g_, and *C*_1_ (=17.44) and *C*_2_ (=51.6 K) are the commonly named “universal” constants of the WFL model (obtained by averaging values for several polymers). Variation of the coefficients *C*_1_ and *C*_2_ for different polymers have been reported in literature as a consequence of the chemical and physical structure of the polymer at the time of experimental determination [61].

The variations in WLF constants have been discussed in terms of the free volume theory [61]; in this framework, *C*_1_ and *C*_2_ are defined as the reciprocal of the fractional free volume at *T*_g_, and the ratio of the fractional free volume at *T*_g_ to the thermal expansion coefficient, respectively, according to Equation (22):

(22) $$log\;a_{T}=\frac{-\left(\frac{B}{2.303}f_{g}\right)\;\left(T-T_{g}\right)}{\frac{f_{g}}{\alpha_{f}}+\left(T-T_{g}\right)}\;$$

where *a_T_* is the WLF shift factor, *B* is an arbitrary constant usually set to 1, *f*_g_ is the fractional free volume at the glass transition temperature, and *α*_f_ is the thermal expansion coefficient above *T*g [61,62].

As storage and processing parameters can be responsible for the variability of WLF constants in food systems, the “universal” constants can promote a considerable error in the calculation of viscosity, being more convenient to calculate the real constants for each system [18,61]. Thus, *C*_1_ and *C*_2_ can be calculated using the method of reduced variables known as the time-temperature superposition principles, where experimental data are reduced to single curves named master curves [18,61].

When compared to the Arrhenius formalism, the WLF model seems more capable of specifying the temperature dependence of viscosity, and to describe that dependency between *T*_g_ and about *T*_g_ + 100 °C [8,18,23,39,41].

The glass transition temperature, *T*_g_ (predicted from the WLF model), is the temperature below which the material changes from the rubbery state (viscous fluid) to the glassy or crystalline state (mechanical solid) during cooling. This property can be experimentally determined by methods such as differential scanning calorimetry (DSC), but the high viscosity of the glassy state (10^7^–10^14^ Pa s) hinders the rheological procedure [8,18,24,41]. The glass transition temperature is frequently considered a reference temperature. As a matter of fact, above this temperature, the difference (*T* − *T*_g_) between storage temperature (*T*) and *T*_g_ is supposed to control the rate of viscosity changes in the food product [24].

The Vogel-Taumann-Fulcher (VTF) model (Equation (23)) also expresses the dependence of viscosity on temperature [8,18,39,58]:

(23) $$\eta =A\exp\left(\frac{B}{T-T_{0}}\right)\;$$

where *A* and *B* are constants of the VTF equation, with *B* calculated as the slope of the linearized form of Equation (23) [18]. In some studies, *T*_0_ is fixed at 184 K, a value estimated from data of aqueous sugar systems of similar concentration [18,38,39]. In other studies, the value used for *T*_0_ is equal to *T*_g_ [39].

Although both equations are interconvertible, literature reports indicated that VTF is more suitable than WFL in sugar systems, since its coefficients are considered “more universal” for employment in different systems under diversified environmental conditions [18].

A Power-law description of relaxation (Equation (24)) is also employed as a semi-empirical model in the temperature dependence of viscosity [8,18,39,58]:

(24) $$\eta =K\;{\left(T-T_{g}\right)}^{m}$$

where *K* and *m* are constants estimated from linearization of Equation (24).

Chemical composition, mainly moisture, sugar content and degree of crystallization, are known to influence honey’s viscosity, but other substances, such as the presence of proteins, polysaccharides and other colloidal material can also contribute to honey’s rheological behaviour.

The water content is a decisive factor varying inversely with viscosity, due to the plasticizing effect of water [11,23,24,26,31,32,33,36,38,40,41,46,51,57,58,63]. The magnitude of rheological parameters decreases with the increasing of the moisture content, due to the role of water in lowering the molecular friction and hydrodynamic forces into the matrix [64]. Water decreases the glassy transition temperature, *T*_g_, due to its capacity to weaken noncovalent interactions, making *T*_g_ a function of moisture and solid content [41].

The above-described dependencies of viscosity on temperature omit the influence of water content in honey, which is a significant drawback as water content exerts a pronounced effect [19]. Thus, two-parameter models, including temperature and water content, came to be used for practical purposes [9,19,22]. References are also found in the literature to combined approaches involving temperature and solids content, shear rate or the Brix degree [15,38,39,65]. Steady, dynamic and creep rheological analysis has also been able to detect honey adulteration by fructose and saccharose syrups [49,51,56,66].

Rheological classification of mono-floral honeys from different floral origins and geographical locations has been achieved by several pattern recognition techniques, including principal component analysis (PCA), cluster analysis (CA), partial least squares (PLS) and support vector machines (SVM) [67]. Recently, adaptive neural fuzzy inference system and artificial neural networks have been used as an alternative to conventional analytical rheological models to evaluate the combined effect of different parameters, such as temperature, shear rate, water content and geographical location, on honey viscosity [64,65,68,69].

## 4. Rheological Measurements

Different rheological devices are used to measure appropriate rheometric flow conditions, reflecting either homo- or heterogeneously structured food materials, and considering the food physical characteristics, applications, type of evaluation, the existence of yielding and slippage, and stream fields associated to the flow in the mouth, while also taking into account the behaviours occurring at the interfaces in food materials [10].

**Figure 2:**
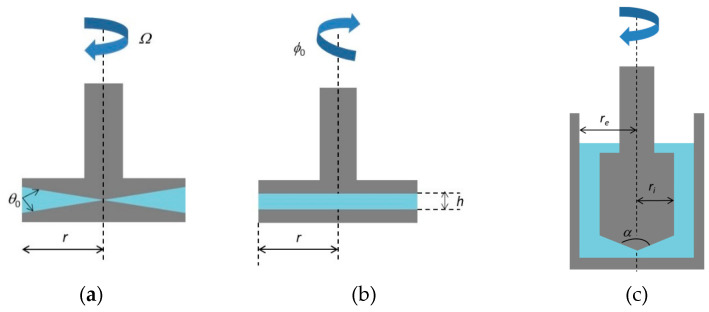
Rheometer geometries: (**a**) cone-plate; (**b**) parallel-plate; (**c**) concentric cylinder.

Steady-state flow tests (constant angular velocity) and oscillatory (dynamic mode) and creep measurements are used to obtain rheological data. Different testing modes employing different geometries (such as disc, cylindrical, parallel plate, cone/plate, T-bar, vane spindles and concentric cylinders) allow the study of bulk materials in rotational rheometers, taking into consideration the characteristics of these materials [12]. Rheometers equipped with parallel plates and cone-and-plate geometries frequently have the capacity to perform amplitude oscillatory shear measurements entailing the application of a sinusoidal stress (or strain) to the upper plate or cone of the device [38] (Figure 2).

Under oscillatory testing, the responses from food materials can be obtained from two regimes: (i) Small Amplitude Oscillatory Shear (SAOS), measuring the linear viscoelastic properties of complex fluids; and (ii) Large Amplitude Oscillatory Shear (LAOS), investigating the nonlinear viscoelasticity [12,39,41,70]. Measurements made under conditions of small deformation allow materials to be probed over supramolecular distances providing relationships between levels of structures and structural organization. On the other hand, the large deformation measurements contribute with a valuable complementary information, principally regarding time-dependent and nonlinear viscoelastic behaviour at large values of stress and strain, which is relevant in food domains [9].

Steady-state tests present a significant drawback arising from the breakdown of food structure, mainly observed at higher shear rate. The non-destructive SAOS analytical method allows overcoming of the mentioned limitations, being suitably employed to explore the rheological properties of semisolid materials without the risk of damage to the molecular structure within the tested food [12]. Beyond the tracking of eventual phase changes in the food materials through processing, SAOS can also clarify the process mechanism [12]. The approved use of SAOS in the food rheological context is based on the firm theoretical knowledge (leading to the development of rheological models for an ample range of frequencies), and capacity to perform appropriate experimental protocols [12,14,70].

The first step in the viscoelasticity characterization is the measuring of the strain amplitude dependence of moduli *G′* and *G*′. A strain sweep is usually performed to establish the extent of the food material’s linearity. Above the critical strain, the network structure of foods is disrupted, and their behaviour becomes non-linear [21,29,60]. Once the linear viscoelastic region (LVR) is defined through the strain sweep test, a food’s structure can be characterized by a frequency sweep test at a strain below the critical value providing understanding about the effect of the interactions forces between colloidal particles [21]. Furthermore, heating or temperature fluctuations applied to a material promote structural changes, which are determined with great sensitivity by temperature sweep tests [29,71]. The oscillation creates deformations small enough to be in the LVR, and the elastic and loss moduli are both independent of the strain amplitude [9,70].

The small magnitude of strain of the SAOS regime is then responsible for the amplitude-independent viscoelastic moduli, and for the sinusoidal character of the stress response. In addition to elucidating the linear viscoelastic characterization, parameters such as the elastic and viscous moduli, *G*′ and *G*′′, respectively (both independent on the shear strain/stress applied), complex viscosity η*, and yield stress lead to information on material functions [12].

Despite the efficiency of SAOS tests for studying the relationship between microstructure and rheology of complex fluids such as food systems, the LVR is observed only for small deformations. As the amplitude of the imposed strain is increased at a constant frequency, a non-linear rheological behaviour is observed, as the relationship between the amplitude of the stress and the amplitude of the deformation of food materials is no longer proportional [12]. Going beyond the linear viscoelastic range, LAOS techniques have been showing great utility in describing the elastic and the viscous properties of complex fluids out of the LVR, which is nearer to the actual processing and function conditions [12,70,72]. Complex classes of fluids exhibiting nonlinear and distorted stress waveforms under LAOS include polymer melts, polymer blends, polymer solutions, block copolymer solutions, block copolymer melts, suspensions, magnetorheological fluids, biological materials, wormlike micelle solutions and food products [70].

With few exceptions (simple liquids or solids), the composition of food products is highly complex, where foods belong mostly to the group of soft condensed matter systems hierarchical nano- and micro-structured [7,9,10,14]. The principal ingredients and their interactions, on a broad range of length and time scales, rule the rheological aspects of these complex products. The structural architecture mentioned is normally due to the presence of proteins and polysaccharides, as well as to the interactions that these macromolecular constituents establish among themselves, and to their interactions with other components of the food system.

Most of the food processing operations present a fast and large strain-deformation, thus requiring a nonlinear rheological approach such as that provided by LAOS [70]. In the nonlinear regime the response is described by the strain amplitude-dependent leading order *G*′(*γ*_0_) and *G*′′(*γ*_0_), and the resulting periodic stress waveform becomes distorted and diverges from a sinusoidal wave [70]. For complex fluids, the leading order LAOS behaviour can be classified by at least four types of strain-amplitude dependence: (i) strain thinning, (ii) strain hardening, (iii) weak strain overshoot, and (iv) strong strain overshoot [70]. LAOS responses can be pictured as parametric curves (Lissajous-Bowditch curves) of the oscillating stress *σ*(*t*) versus strain *γ*(*t*) or stress *σ*(*t*) versus shear rate $\dot{\gamma }$(*t*).

Temperature and duration of measurements considerably influence foods rheology. In this context, the technique Time-Temperature Superposition Principle (TTSP) enables an extension of the frequency regime at a reference temperature of the testing material [16,54,73]. The TTSP, which has been extensively applied to homogeneous liquids, establishes the basis of accelerated aging procedures. It assumes that the short-term viscoelastic behaviour at higher temperatures is analogous to the long-term behaviour at some lower reference temperature [54], being formally written as (Equation (25)):

(25) $$G^{*}\left(\omega ,T\right)=\frac{G^{*}\left(\omega a_{T},T_{0}\right)}{b_{T}}\;$$

where *T*_0_ is the arbitrarily chosen reference temperature; *a_T_* and *b_T_* are the frequency (horizontal) and moduli (vertical) shift factors, respectively, and their temperature dependence can be expressed as the Arrhenius model, WFL model or polynomial equations. In Equation (25), G* can be replaced by any other rheological property.

In this approach, the temperature only shifts the time scale of the relaxation process without altering its nature, or the structure of the polymeric liquid [54,73]. Frequency-dependent data at different temperatures can be superimposed by the simultaneous horizontal and vertical shifts along a logarithmic time scale (for both axes), generating a predictive master curve. The TTSP can be successfully applied to liquid-state honeys, as *G′* values of honey samples at different temperatures can be overlapped into a master curve employing a reduced frequency [16,54,62].

## 5. Rheological Properties of Honey

Steady shear and dynamic shear assays have been performed to analyse the rheological properties of honey of different botanical sources and origins, either in its liquid state or in its crystallised form [2,8,11,15,18,23,24,27,31,32,34,35,37,38,39,43,45,50,54,64,68,74,75]. Additionally, parameters calculated from oscillatory measurements are very susceptible to physical and chemical changes, justifying their usefulness in the rheological evaluation of honey.

The main targets of honey rheological studies have been the geographical and/or botanical differentiation, authentication/adulteration analysis, the presence of additives, or the influence of sugar composition, moisture content, storage time, temperature and preservation techniques in the processing stages, as well as crystallization events. Table 1 summarizes some of the main contributions to rheological behaviour characterization of honey from different countries and botanical origins.

Based on rheological properties of six Greek honeys, two honeydew honeys (pine and fir) and four uni-floral nectar honeys (thymus, orange, helianthus and cotton), Yanniotis et al. [31] found that the samples showed Newtonian and time-independent behaviour and that their viscosity was more sensitive to temperature changes at low moisture contents (after the Arrhenius model fitting to data). Pine and fir honeys, presenting higher concentrations of di- and tri-saccharides, displayed the highest values of viscosity at each moisture content and temperature, followed by thymus honey. Observed differences in viscosity among the six honeys may have arisen from the presence of colloid materials, also reflecting the relevance of botanical source on honeys’ viscosity.

A non-Newtonian shear-thinning and time-independent behaviour was observed in Spanish honeys from Galicia (with water content ranging from 16.89% to 17.67%, and °Brix of 81%), at 25 °C, by Gómez-Diaz et al. [2], and in all cases when low values of SR were applied. The flow characterization of Galician honeys demonstrated the decrease of apparent viscosity with the increment of SR, well described by the Ostwald-de-Waele (Equation (2)), and the direct influence of water and sugar content on the viscosity. Later, the same authors [58] analysed the influence of temperature on the viscosity of several Galician honey samples, employing the Arrhenius (Equation (20)), Power-law (Equation (24)), WFL (Equation (21)) with varying *C*_1_ and *C*_2_ parameters, and VTF (Equation (23)) models. The temperature effect was stronger in the low temperature range, which turns this effect into a relevant issue when the operation temperature is under 25 °C. Since WFL and VTF models include the *T*_g_ in their equations, this parameter was experimentally determined by DSC. Values of *T*_g_ (near to −40 °C for the studied samples) decreased as the water content in honey increased, in a similar way reported by Lazaridou [24]. The four models were adequately fitted to experimental data, although the Arrhenius model proved to be the best model.

According to Recondo et al. [18] in their study of the viscosity-temperature dependence of uni-floral honey from Argentina and supersaturated solutions of glucose, fructose and glucose/fructose (at 1.22 ratio), all models described the experimental behaviour quite satisfactorily, although no extrapolation could be made outside the limited range for which the models’ coefficients were calculated. For the Argentinian honey, VLF, VTF, and P-L models (Equation (21), (23) and (24), respectively) fitted better than the Arrhenius one. Moreover, the extrapolation of fitted curves into the glass transition region showed that the Arrhenius model predicted the lowest temperature dependence whereas the WLF model (with coefficients calculated by the reduced variables method) predicted the highest viscosity values close to *T*_g_. On the other hand, VTF and P-L models provided curves with intermediate solutions between Arrhenius and WLF models. According to Recondo et al. [18], since P-L equation is mathematically undefined for *T* = *T*_g_, and its coefficients lack physical meaning, VTF appeared as the best alternative to predict viscosity in the case of any extrapolation.

For the Australian honeys (mostly *Eucaliptus*) studied by Sopade et al. [8] in the range 2–40 °C, the WLF was the most satisfactory model; its estimated constants *C*_1_ and *C*_2_ were calculated through a nonlinear regression analysis and generated different values from the “universal” values (Table 1), but very similar to those obtained by Recondo et al. [18]. Alongside the Arrhenius model, Lazaridou et al. [24] also found that WLF was very suitable for modelling the rheological behaviour of Greek honeys with temperature, either using fixed “universal” constants, or allowing *C*_1_ and *C*_2_ to vary.

Smanalieva and Senge [30] analysed the rheological and physicochemical properties of five German uni-floral honey (false acacia, heather, sunflower, lime and rape) to establish the relation between the material properties and the botanical origin. Changes in the temperature and in the crystalline state allowed the validity of Newton (Equation (1)), Power-law (Equation (2)), and Herschel-Bulkley (Equation (3)) constitutive equations on steady shear experimental data. All samples were in a crystalline state, except the false acacia honey which exhibited a Newtonian behaviour. The non-Newtonian behaviour of the crystalline honeys was described by the Power-law model from 30 to 50 °C. In the range 10–20 °C shear stress–shear rate data were better described by the Herschel-Bulkley model, since the presence of crystals in honey samples caused the occurrence of a yield stress [30]. The results of the selected German honeys confirmed the dominant liquid-like property in the temperature range 0–75 °C, except for the heather honey which depicted a viscoelastic profile (*G*′ > *G*′′).

Witczak et al. [32] observed a non-Newtonian behaviour of heather honey samples from south Poland. Results highlighted the non-Newtonian shear-thinning characteristics of the investigated honeys, with tendency to yield stress and thixotropy (the latter confirmed by the negative values of Weltman’s *B* parameter). Shear-thinning could be attributed to the large amount of high molecular compounds such as proteins in heather honey [21,23,32,33]. The applicability of the Herschel-Bulkley model resulted in parameter dependent on the type of honey sample, its moisture content, and the measurement temperature. The yield stress and the Herschel-Bulkley consistency were the highest for heather honey samples containing the lowest amount of water, and the flow index behaviour confirmed the shear-thinning character [32]. Arrhenius *E*_a_ values depended on water content but could also be affected by the content and hydration degree of protein substances accountable for the formation of honey pseudo-gel structure [32], and eventually by the presence of individual mono-, di- and tri-saccharides. Temperature also reduced the area of the hysteresis loop, confirming the dependence between temperature and thixotropy observed by Smanalieva and Senge [30].

Parameters *K*′ and *K*′′ from the P-L relationships (Equations (10) and (11)) applied to experimental data were also found to be temperature-dependent and affected by water content [32]. Due to the non-Newtonian nature of the studied samples, the Cox-Merz rule (Equation (13)) could not be observed, and η* correlated well with the apparent viscosity by means of the Power-law relationship (Equation (15)), which depended on the variety of honey.

Samples of Romanian linden, black locust, rape, sunflower, honeydew and multifloral honeys were examined regarding the relationship between the rheological properties and the sugar composition [21]. All samples had glucose, fructose, maltose, and sucrose, and in honeydew samples melezitose and trehalose were also present. A high tan*δ* was obtained (Table 1), demonstrating that honey particles were highly un-associated. Variations in pollen composition, sugar percentages and water content were responsible for the differences observed in both *G*′ and *G*′′ of all honeys; viscosity of these honeys were mostly influenced by glucose and fructose. On the one hand, sunflower honey, which was found to be Newtonian (as well as black locust, linden and multifloral honeys), displayed the lowest average fructose/glucose (F/G) ratio (=1.0), and crystallized quite rapidly after the evaluation of the rheological behaviour due to its highest sugar content. On the other hand, rape honey (with the highest content of carbohydrates, namely glucose) and honeydew honey (with a large number of high molecular compounds such as proteins, and a minor reducing sugar content) showed non-Newtonian shear-thinning behaviour with thixotropy. Lastly, a linear discriminant analysis managed to correctly classify 78.8% of the honey samples and predict viscosity based on the carbohydrate composition and the rheological properties *G*′′ and *σ*.

The six types of Tunisian honey from several floral origins (eucalyptus, orange, rosemary, thyme, mint, and horehound), studied by Boussaid et al. [33] were classified as non-Newtonian at shear rate range of 0.01–500 s^−1^ and 20 °C. In this study, whose results agreed with other published in literature for the non-Newtonian honeys [2,21,32,59], the observed behaviour was shear-thinning. The Herschel-Bulkley model (Equation (3)) provided the best fit allowing the yield stress to be calculated, a parameter related to the presence of sugar crystals in honey [30,32,33]. Thyme honey had the highest value of yield stress (Table 1), also presenting the highest consistency coefficient and the lowest water content, because of the anti-plasticizing effect of sugars (in opposition to water). Contrary to horehound honey, which depicted the lowest values of Weltman’s parameters *A* and *B* (Equation (6)), thyme honey contributed to an increase in initial stress (*A*), due to the formation of a more stable sugar network [33], and the highest *B* (Table 1). In all honeys, *B* presented negative values indicating a thixotropic behaviour.

As usually observed, viscosity decreased with increase in temperature, but heating did not affect the shear-thinning behaviour. Thyme honey presented the highest Arrhenius *E*_a_ value, which may be associated with many inter- and intra-interactions between sugar chains [33]. The viscous nature of honey samples was confirmed by the relation *G*′′ >> *G*′ in the whole frequency range, maintaining the trend in the 20–50 °C, and considering that no crossover point was detected for the two moduli.

Some Brazilian honeys also behaved as non-Newtonian fluids. The example is given by Maieves et al. [43] who studied Brazilian mono-floral honeys from *Hovenia dulcis* flowering produced by *Apis mellifera* (four samples) and *Tetragonisca angustula* bees (one sample), very prone to fraud as they are only available in specialized markets and usually sold for higher prices than the most usual types of honey. As expected, the sample with the highest *a*_w_ presented the highest moisture content, the lowest apparent viscosity (*η*_app_) at all temperatures, and the lowest *E*_a_ value. The Power-law adjustment gave rise to interesting results: all samples were Newtonian or almost Newtonian at 30 °C, becoming shear-thinning at higher temperatures. However, at 40 and 50 °C, Maieves et al. [43] observed a shear-thickening behaviour of the sample from the *T. angustula* stingless bee, which turned to a shear-thinning behaviour at 60 °C.

Travnicek et al. [46] investigated the rheological behaviour of three selected Czech honeys (blossom-honeydew honey, blossom-honeydew lime honey, and blossom honey-nectar from plants blooming in spring). The authors studied the dependence of shear stress on shear rate, and the dependence of dynamic viscosity on temperature. Although a higher content in water generally corresponds to a lower honey viscosity (as already mentioned), this was not observed in this study [46]. Temperature dependence on dynamic viscosity was discussed based on the Arrhenius model (Equation (20)), allowing the calculation of activation energy *E*_a_ whose values agreed with the ones reported by other researchers for similar types of honey (apart from honey samples with high content of macromolecular substances leading to a thixotropic behaviour). In addition, the highest activation energy value matched the highest viscosity value.

Oroian et al. [38] investigated the rheological properties of six Spanish honeys in liquified state (from mono-floral, poly-floral and honeydew varieties) at different conditions of temperature and concentration, also correlating it with physicochemical characteristics. The studied honeys showed a Newtonian behaviour, and their viscosity decreased with temperature and increased with the solid content. Activation energies computed from the linear Arrhenius plot increased with increasing viscosity in a similar pattern as that reported in the literature for honeys from difference provenance; the same happened with parameters of the VTF model determined by nonlinear regression [38]. Honeydew, with the lowest water content and the highest solid content (°Brix), exhibited the highest viscosity, as opposed to the mono-floral rosemary honey presenting the highest water content, the lowest °Brix and the lowest viscosity (Table 1). The authors proposed a simplified model (Equation (26)),

(26) $$\eta =1.9\;\cdot \;10^{-17}\exp\left(0.134C+\frac{8818.5}{T}\right)$$

To interpret the combined effect of temperature and solid content on the viscosity of Spanish honeys, corresponding to activation energy, values were similar to those reported in Newtonian foods such as clarified cherry juice and grape pekmez [38].

Still considering Spanish honeys, Oroian et al. [54] measured the viscoelastic parameters, *G*′ and *G*′′ moduli in the 5–40 °C range, using oscillatory thermal analysis, aiming to obtain a model for describing viscoelastic behaviour along with temperature. The viscoelastic parameters *G*′ and *G*′′ increased with frequency, contrary to the complex viscosity η* which was frequency-independent. All the viscoelastic parameters were positively and negatively influenced by the °Brix and the moisture content, respectively. As magnitudes of *K*′′ (viscous intercept) were much greater than those of *K*′ (elastic intercept), the Spanish honeys showed a liquid-like behaviour, frequency-dependent, which is consistent with the magnitude of the complex viscosity [54]. Moreover, *K*′ and *K*′′ increased with increasing moisture content and decreased with increasing temperature. As *G*′′ was much higher than *G*′, the temperature effect on dynamic rheological properties was only characterised by *G*′′. The increase in kinetic energy with temperature led to a decrease in viscosity and, consequently, to a decrease of the viscous response. With the application of TTSP, an enlargement of the frequency range of the mechanical spectrum of a solution was achieved [54]. A mathematical model of the viscoelastic functions, which depended on *G*′′ values, was developed to determine the vertical shift factor, *b_T_* (Equation (25)), and this approach was based on a fractional Maxwell model. For all the studied honey samples, the vertical shift factor showed a huge magnitude. Given the fact that *b_T_* could be explained by density and temperature product changes, a 4th order polynomial equation was obtained (Equation (27)):

(27) $$b_{T}=1+\left(3\alpha +\frac{1}{T_{0}}\right)\Delta T+\left(3\alpha^{2}+\frac{3\alpha }{T_{0}}\right)\Delta T^{2}+\left(\alpha^{3}+\frac{3\alpha^{2}}{T_{0}}\right)\Delta T^{3}+\frac{\alpha^{3}}{T_{0}}\Delta T^{4}\;$$

where *T*_0_ is the reference temperature and *α* is the thermal expansion coefficient. Equation (27) provided a better fitting than the Arrhenius one. The high accuracy of fit justified the appropriateness of the TTSP model (Equation (25)) to extrapolate the dynamic viscoelastic properties, regardless of the botanical origin of honey (mono-floral, poly-floral, or honeydew). The validity of the model was checked by relaxation tests operated at all the temperatures used at oscillatory assay. The relaxation and the retardation activation energy, as well as the relaxation modulus (with the same polynomial equation evolution at all temperatures) obeyed the proposed model, which was based on vertical shift factor (*b_T_*) computation [54]. Since the two activation energies had the same magnitude, just one of them could be selected. As expected, activation energy was a function of the moisture content–temperature.

Taking into account the strong influence of temperature, moisture content and frequency on the Spanish honeys, Oroian [68] used artificial neural networks (ANN) and an adaptive neuro-fuzzy inference system (ANFIS) as predictive tools of the viscoelastic parameters of nine different types of Spanish honey samples [68]. As previously observed [38,54], a Newtonian behaviour was also detected; the loss and the storage moduli magnitudes were substantially influenced by frequency, contrary to what was observed with the complex viscosity [68].

The TTSP approach overcomes the experimental difficulties (the restrictions of viscometers/rheometers or the physical integrity of samples) expected for the rheological study over broad operating ranges (such as SR, frequency, and temperature). Based on the master curve generated from the TTSP methodology (Equation (25)), a detailed picture of the physicochemical and viscoelastic properties of Tulsi, Alfalfa and two types of Manuka honey (obtained from different medicinal plants with anti-inflammatory activity) was provided by Nguyen et al. [62]. *G*′ and *G*′′ were investigated, regarding their variation, with sub-zero temperatures (from −65 to −15 °C). Up to *T*_g_ of each honey, a sharp increase in both moduli occurred (Table 1); in this glass transition region, *G*′′ dominates over *G*′. Modulus traces crossover at the low end of the temperature range, remaining constant, and the elastic response dominated over the viscous; this is recognized as the glassy state where the motion of molecules is restricted. Within the temperature range of the glassy state, a linear correlation between temperature and log *a*_T_ was verified for these honeys, suggesting that solidified honey obeys a modified Arrhenius equation where *E*_a_ values (Table 1) were comparable to carbohydrate matrices with glassy consistency (glucose syrup, for example). At the upper temperature range of the glass transition, *a*_T_ dependence on temperature was better modelled by the WLF equation within the theoretical framework of free volume (Equation (22)), and data fitting parameters related to this equation were for the first time given in this investigation. Estimates of the fractional free volume at the glass transition temperature were about 0.040 for all honeys, denoting that that honey matrix has entered a state of kinetically trapped equilibrium in molecular relaxation [62]. It should also be noted that values of the mechanical glass transition temperature matched those determined from calorimetric *T*_g_ (Table 1). With the lack of 3D polymeric structures in honey, it is the sugar molecules that govern the vitrification patterns for the calorimetric and the rheological techniques [62].

The Polish honeys studied by Juszczcak et al. [23] belonged to five nectar varieties (acacia, buckwheat, linden, multifloral and rape), a nectar-honeydew and a honeydew variety, and exhibited a Newtonian behaviour. Nectar-honeydew honey (with the lowest moisture content) presented the highest viscosity value at all temperatures and the highest activation energy. Values of *η*_g_ (falling into the viscosity range of the glassy state) and *T*_g_ were similar to those of Al-Malah et al. [35] for Jordanian dark-coloured honeys. Apparent flow activation energy, computed from WLF equation, decreased with increasing temperature, and its highest values were observed for nectar-honeydew honey.

Considering the growing importance of honey in Burkina Faso, Escriche et al. [15] performed the chemical characterization and the rheological evaluation of samples from different locations of the country, Kampène, Bouroum-Bouroum, and Passena. Burkina Faso honey presented a higher viscous nature (*G*′′ >> *G*′) and the three viscoelastic parameters *G*′, *G*′′ and η* showed a strong dependence on temperature. The Newtonian behaviour was demonstrated by the independency of η* on the frequency, following a pattern observed with other Newtonian honeys. Moreover, phase angle (*δ*) values were closer to 90 ° at all temperatures, corroborating the Newtonian response. Values of *E*_a_ were obtained for the complex viscosity—temperature and the loss modulus—temperature data (Table 1). The Burkina Faso honey sample with the highest moisture content presented the lowest *E*_a_ value. This correlation also justified the lower value of *E*_a_ for Burkina Faso honey when compared to those of honeys from other geographic locations [23,25,32,38,43]. The combined influence of the temperature and concentration (76–81 °Brix) on honey’s rheological properties permitted the suggestion of Equations (28) and (29) regarding the evaluation of the complex viscosity and the loss modulus, respectively [15]:

(28) $$\eta^{*}=3.98\;\cdot \;10^{-17}\exp\left(0.275C+\frac{42.003}{RT}\right)\;$$

(29) $$G\prime \prime =4.22\;\cdot \;10^{-17}\exp\left(0.292C+\frac{4.178}{RT}\right)$$

Third degree polynomial equations were proposed to predict η* and *G*′′ (*r*^2^ > 0.98), in conformity with honey chemical composition and temperature (Equations (30) and (31)):

(30) $$\log\;\eta^{*}=0.59-1.53\cdot X_{1}+1.36\cdot X_{2}-0.34\cdot X_{7}+0.05\cdot X_{7}^{2}+0.13\cdot X_{1}\cdot X_{7}-0.10\;\cdot X_{2}\cdot X_{7}-0.16\cdot X_{7}^{3}+0.38\cdot X_{1}\cdot X_{7}^{2}-0.33\cdot X_{2}\cdot X_{7}^{2}+1.27\cdot X_{3}\cdot X_{7}^{2}+1.07\cdot X_{4}\cdot X_{7}^{2}$$

(31) $$G\prime \prime =1.39-1.58\cdot X_{1}+1.41\cdot X_{2}-0.40\cdot X_{7}+0.05\cdot X_{7}^{2}+0.50\cdot X_{1}\cdot X_{7}-0.47\;\cdot X_{2}\cdot X_{7}-0.07\cdot X_{7}^{3}+0.91\cdot X_{1}\cdot X_{7}^{2}-0.85\cdot X_{2}\cdot X_{7}^{2}$$

The seven design variables comprised fructose (*X*_1_), glucose (*X*_2_), sucrose (*X*_3_), sugars (*X*_4_), moisture content (*X*_5_), non-sugar substances (*X*_6_) and temperature (*X*_7_). Polynomial modelling was not influenced by the moisture content, nor by the non-sugar substances. As for the other variables, glucose revealed a linear positive influence on η* and *G*′′, while fructose and temperature exhibited an opposite trend [15].

Another developing country with a potential to be explored in terms of the economic and environmental importance of honey is Mozambique. Escriche et al. [69] examined several samples from three provinces of Mozambique, including physicochemical and rheological analysis. Rheological parameters *G*′, *G*′′ and η* showed a similar dependency pattern towards the temperature and frequency observed earlier in Newtonian honeys from other countries, and honeys were found to have a more pronounced viscous nature. Artificial neural networks (ANN) were used to predict the rheological parameters based on five inputs (temperature, frequency, moisture content, glucose and fructose), and built on the multilayer perceptron (MLP), probabilistic neural network (PNN), recurrent neural network (RNN), and modular neural network (MNN) classifier methods to create the pattern recognition systems [69]. PNN was found apt for *G*′, whereas MLP was the best model for *G*′′ and η*. However, *G*′ was modelled with a lower regression coefficient (*r*^2^ = 0.758) when compared to the other two viscoelastic parameters (*r*^2^ = 0.950), probably due to its high sensitivity to any particles in suspension (including pollen grains, sugar, and glucose crystals). The work developed by Escriche et al. [69] proved that for these honey samples from Mozambique (mostly honeydew, following the criteria of colour and conductivity), frequency and moisture were the most relevant factors for *G*′ and *G*′′. Conversely, glucose and fructose had less influence than moisture for *G*′, and the smallest influence of all inputs for *G*′′ and η* [69].

Crystallised honey is thus a two-phase structure of a semi-solid type whose consistency is dictated by the mass fraction of both the solid phase and the morphology of the crystalline structure [11]. To shape the semi-liquid consistency of honey is a challenging issue, of academic and industrial interest. With a controlled crystallization, through a cooling process between 14 °C and 18 °C, a creamed honey can be created [29,47], meeting consumer preference. A higher viscosity can be detected when crystallization rate is low and the fewer core crystals lead to a layered structure [47]. The same happens when honey is liquefied after heating at 70 °C, and the recrystallization is likely to form agglomerates across the container [11,47].

During processing, distribution, handling and storage, honey is subjected to repeated thermal changes that affects its stability and its rheological characteristics. In the case of creamed honey, thermal stability is crucial for its quality in terms of consumer acceptance. However, temperature sweep tests (*G*′ and *G*′′ as a function of time) do not provide accurate information on repeated thermal changes [29]. Karasu et al. [29] used a thermal loop test, carrying out eleven thermal cycles over a temperature range (5–50 °C), to evaluate structural changes and the thermal stability of creamed honey, when investigating its rheological properties. SS-SR curves and the flow index behaviour *n* (from Power-law model, Equation (2)) values indicated a non-Newtonian shear-thinning behaviour, probably due to the presence of crystals and the large number of high-molecular-weight constituents such as protein and dextran. Following a trend already reported for natural honey, the increase in temperature causes a thermal expansion and a reduction of intermolecular forces, accountable for the decrease in *η*_app,50_ (apparent viscosity calculated after temperature sweep tests carried out at 50 s^−1^). The variation of *η*_app,50_ with temperature followed a non-Newtonian trend in the studied temperature range. At 10 °C, and for the first 80 s, *η*_app,50_ increased with increasing temperature, as moderate temperatures (10–21 °C) probably promote crystallization. However, after that initial shearing time, *η*_app,50_ decreased with temperature, which could be attributed to a structural breakdown leading to a deformed crystallization structure. At 25 and 40 °C, *η*_app,50_ always decreased with shearing time, suggesting that warmer temperatures (21–27 °C) do not encourage crystallization which is probably inhibited over 27 °C [29].

From upward followed by downward shear rate sweeps, hysteresis loops were formed enabling the evaluation of thixotropy, and results were consistent with those reported in the literature for other honeys [29,30,32]. The explanations for the observed thixotropy may lie in the higher rate of disentanglement of macromolecules (when compared to that of re-entanglement) and in the presence of colloids (detected in honeys such as buckwheat, heather and white clover honeys [29]). As Smanalieva and Senge [30] reported, hysteresis can also result from the distinct dissolving processes under heating and supersaturation and nucleation crystal behaviour under cooling conditions [30].

Considering the temperature sweep test from dynamic shear measurements, *G*′′ >> *G*′ over the angular frequency range, indicating a more viscous nature for the creamed honey at all working temperatures. The complex viscosity values, reported in the literature to be frequency independent in the case of different honey types, depicted here a slight decrease with increasing frequency [29]. Dynamic parameters *G*′ and *G*′′ decreased with increasing temperature and obeyed to Arrhenius model. The creamed honey studied by Karasu et al. [29] presented a high *G*′ value at the lowest temperature (10 °C) which corresponded to a poor spreadability, mirrored in the lack of a linear positive correlation with frequency at 10 °C. Regarding the thermal loop results, the relative structural index, Δ, determined from the maxima values of all cycles, for *G*′ and *G*′′ (Equation (32)),

(32) $$\Delta =\frac{G_{\max}^{\prime }\left(G_{\max}^{\prime }\right),i}{G^{\prime }\;\left(G^{\prime }\right),1};i=1\;to\;15\;$$

decreased with the number of cycles. It is expected that a small value for Δ at the end of the thermal cycle corresponds to the smallest increase in *G*′, and consequently to the highest thermal stability [29,76]. Besides, when Δ is close to unity, a material structure is considered less influenced by thermal stress [29]. Given this background, results from Karasu et al. [29] suggest that creamed honey had low thermal stability, showing a great structural change due to the thermal stress applied in the 5–50 °C range and reflected in the variations of *G*′ and *G*′′ with time in the thermal loop analysis. Heating and dissolution of crystals during thermal loops led to the loss of the crystalline structure and to irreversible rheological changes [29].

Heating, which also facilitates honey handling and packaging, and eliminates microorganisms, influences the viscosity values of honey [40]. The retardation of the crystallization process in honey can be accomplished by ultrasound (US), an advantageous method when compared to an expensive and time-consuming heating treatment (HT) that degrades honey quality and its sensory attributes. In the work developed by Kabbani et al. [13], crystallized rosemary honey samples were liquefied in a US bath of 40 Hz at a temperature range of 40–60 °C, for time intervals between 20 and 60 min. The effect of US on honey liquefaction was evaluated through rheology and crystal content, and compared with liquefaction by HT, in the same temperature and time experimental conditions. Viscosity values for samples with (lower) and without (higher) US-treatment showed a Newtonian behaviour (as reported in the literature for light-coloured honeys such as rosemary honey). In this comparison process, where US treatment was found to provide a faster liquefaction (without the need to increase the temperature up to 50 °C or higher), flow behaviour was discussed in terms of the temperature variation, at a constant SR and during a time range. Flow activation energy values, obtained by the Arrhenius model (Equation (20)), were lower for the US-treated samples, evidencing a lesser sensitivity to temperature change when compared to the HT-treated ones. However, these results did not consider the influence of sugars, moisture content, colloids and other components present in the investigated honey [13].

Rheometric analysis, performed on three types of Polish honey defined by a varied crystalline structure (rape, multi-floral, and buckwheat), allowed Bakier et al. [11] to identify the rheological properties of honeys in the liquid and crystallised states. In the case of crystallised honeys, a shearing process was carried out in order to obtain samples of honey with identical deformation history [11]. The Newtonian model was fitted to the steady shear state experimental data regarding liquefied honeys. On the other hand, the Ostwald-de-Waele model (Equation (2)) predicted crystallised honey’s flow curves built from shear stress–shear rate experimental data, enabling the determination of consistency coefficient, flow index, and the dependence of apparent viscosity on shear rate (Table 1).

Regarding the oscillatory assays, the liquefied honeys presented a linear dependence of the complex modulus on the frequency; rape honey exhibited the highest values of *η* (correlated with water content), G* and η*, as well as the fastest linear decrease of *δ*. As for the crystallised samples, rape honey depicted the highest values of shear stress (at both increasing and decreasing shear rates) and apparent viscosity, due to the morphology of the crystalline structure (reflecting a narrower crystal size distribution when compared to the other two honeys). The impact of time on rheological properties was more pronounced in rape and multifloral crystallised honeys, as the surface areas of the hysteresis loop were comparatively larger than that of buckwheat honey; this effect is related to the breakup of crystalline agglomerates and the framing of the velocity profile in the rheometric flow [11].

The desirable rheological properties of honey can, therefore, be developed based on the modelling of its structure, which is feasible through the crystallization process. In addition to modelling consistency and utility in the field of the hydraulic transport of honey, the results contribute to the analysis of the sensory perception of crystallized honey, given the oscillatory nature of chewing.

Due to its high market value, honey is often adulterated with less expensive industrial sweeteners which mimic its sugar profile [3,49,51,56,60,77,78,79]. Common adulterants in honey include starch syrups (high fructose corn, corn, rice), inverted syrups (for example, inverted syrup from sugar cane/sugar beet, and jaggery), starch or inverted syrups fed to bees (providing honey with a high level of indirect sugar), or even low quality honey added to high priced honey (for example, acacia honey adulterated with rape honey, or heather honey adulterated with common honeys) [45,49,51,56,66,77,79].

Detection of direct or indirect adulteration is thus mandatory for authentication purposes and encompasses several analytical approaches, such as thin-layer chromatography (TLC), stable carbon isotopic ratio analysis (SCIRA), gas chromatography (GC), high-performance liquid chromatography (HPLC), high-performance anion-exchange (HPAEC), infrared-based spectroscopy (IR), Raman spectroscopy, nuclear magnetic resonance (NMR), electronic tongue, the metabolomics-base detection approaches (such as ultra-performance liquid chromatography coupled with quadrupole time-of-flight mass spectrometry, UPLC-Q-ToF-MS) and DNA metabarcoding [49,51,56,60,66,77,78,79].

When compared to the previous methods, the determination of the ratio between or among chemical elements, as principal components, supposing these ratios are a constant, is an advantageous method in terms of time, price, and sample preservation [78]. Thus, adding any amount of a substance into honey should alter the ratio of constituents or produce an anomaly in the composition. In this context, rheological characteristics of honey may exemplify a useful approach, as physical properties are affected by the F/G ratio of honey and evidently by its crystallization rate [19,21,49,51,56,79]. Crystallization of honey usually occurs when F/G ratio is higher than 1.33. Glucose, which tend to crystallize more by virtue of its lower solubility, may exist as α-D-glucose monohydrate (crystallization below 50 °C), α-D-glucose and β-D-glucose anhydrous forms (stable at 50–80 °C and above 80 °C, respectively) [19,80]. Glucose transition temperature from monohydrate to anhydrous forms is reduced to below 30 °C when saturated with fructose. From the rheological point of view, crystallized honeys usually exhibit a non-Newtonian flow behaviour with yield stress and thixotropy, evidencing thus a remarkable dependence on temperature [29,30,56,79]. Therefore, F/G ratio and storage temperature appear as crucial factors for crystal size formed in the honey.

The spontaneous precipitation of glucose at room temperature during storage affects the quality of honey and consumer acceptability. The rate of time dependent crystallization is controlled by water content, presence of nucleation seeds (resulting from the monohydrate form of glucose), degree of supersaturation and viscosity [80,81]. For some mono-floral honeys homogeneous crystallization is a natural event; but for most commercial honeys the formation of crystals may result in phase separation, sedimentation and to the augment of water activity up to levels probably in accordance with microbial fermentative processes [80]. Modifications in rheological properties of honey resulting from crystallization have been described in the literature [47,80,81,82].

Crystals of honey, mainly glucose monohydrate, represent an industrial problem concerning the handling and processing in machinery due to the increase in viscosity. The delay of the crystallization phenomena can be induced by thermal processing at 50 °C, which reduces the crystal count and viscosity [82]. However, liquefaction by thermal treatment leads to several harmful reactions and to the formation of food contaminants such as 5-hydroxymethylfurfural (5-HMF) even beyond the legal limit [81,82]. Önür et al. [82] studied three Turkish honey varieties (sunflower, cotton, and canola) undergoing different treatments (thermal, ultrasound and high hydrostatic pressure), and evaluated the alterations detected in the physicochemical properties and the formation of 5-HMF. Sunflower honey exhibited a non-Newtonian shear-thinning profile in the crystallized form. After liquefaction by US technique, sunflower honey samples showed Newtonian behaviour, allowing the authors to conclude that the distinct rheological behaviour could be attributed to the differences in the solid content (mostly sugars) and to the effect of US power intensity [82].

Rheological analysis combined with multivariate data analysis and machine learning techniques have been performed to predict rheological properties as a function of several variables (temperature, frequency, chemical composition) and to forecast the botanical and geographical origin of honey, for the purpose of authenticity certification [51,56,64,68,69,71,75]. Yilmaz et al. [56] undertook the first rheological study on detection of honey adulteration, investigating the steady, dynamic shear and creep behaviour of natural Turkish honey adulterated with saccharose and fructose syrups at different ratios (0–50% *w*/*w*). Rheological results were confirmed by HPLC-RID analysis, enabling the correlation to be established between rheological parameters and sugar composition. The evaluation of the steady-state shear properties pointed to a Newtonian flow behaviour for all the honey samples, from the natural (control) honey to the adulterated ones, with fructose and saccharose syrup addition decreasing the viscosity of natural honey [56]. Viscosity of all samples decreased with increasing temperature (as the thermal energy of molecules increased), but these findings did not devaluate the referred relationship between adulterants and honey viscosity [56]. In fact, not only can steady shear rheological analysis be used to detect honey adulteration, but it also allows to establish the temperature range for this detection. In addition, results from this work showed that samples adulterated with 10% sucrose and fructose syrups could be detected between 5 and 20 °C. On the other hand, dynamic shear analysis showed that *G*′ and *G*′′ values of adulterants and adulterated samples increased with frequency in a non-linear and linear relationship, respectively, expressing the relevance of the loss modulus in the discussion of adulterated honey viscoelasticity. Viscous characteristics prevailed in all analysed samples, as *G*′′ magnitude was notably higher than that of *G*′, indicating a liquid-like behaviour. According to the results, *K*′′ showed the potential to be a good indicator of honey adulteration at levels in the 10%–50% range [56]. As mentioned before, saccharose/fructose syrup and honey tend to crystallize since highly concentrated sucrose solutions might be in a metastable state. As shearing may eventually lead to a faster crystallization altering the rheological properties of the studied samples, authors carried out creep and recovery tests. The Burgers model (Equation (19)), which gives information about the internal structure of a food system, was applied enabling the display of an initial elastic response, a viscoelastic behaviour and a Newtonian type of flow [7,56].

The creep parameters, namely *G*_0_, *G*_1_ and *η*_1_ (Equation (19)), did not show a response pattern regarding the increase in adulterant level, except for the Maxwell dashpot parameter which decreased as the adulterant level increased. Moreover, the values of creep and recovery compliance *J* as a function of time were higher for adulterated honey samples presenting higher saccharose/fructose syrups. The results seem to imply that the addition of these adulterants prompted a large deformation in honey viscoelasticity, impairing its internal structure for the same tested stress [56]. No recovery was noticed for adulterated honey samples after the force was removed, which was consistent with the Newtonian behaviour observed during the force application. Nonetheless, the recovery start point increased as the adulterant level increased. Hence, the results led the authors to consider that creep-recovery analysis was efficient to detect the changes operated in the creep-recovery performance of natural honey caused by adulteration, and the consequent deformation of honey structure. Expressive correlations between rheology parameters and sugar composition of adulterated honey samples led to the assumption that these parameters could be used as a combined criterion to detect adulteration at specified ratios. PCA was also performed to classify natural and adulterated honey based on rheological and physicochemical results, confirming the potential use of K*, *K*′′, *η*_0_ (Equation (19)) and *η* to detect the investigated adulteration.

Oroian et al. [51] also evaluated the influence of adulteration agents (glucose, fructose, inverted sugar, hydrolysed inulin syrup and malt wort) on the rheological properties of an authentic honeydew honey, using steady state, dynamic state, and creep test. The honeydew honey and the adulteration agents behaved as Newtonian fluids. The adulterants that most influenced dynamic viscosity (*η*) were fructose (decreasing it), and glucose and hydrolysed inulin (increasing it). Regarding thixotropy, the addition of fructose contributed to the decrease of the thixotropic area, contrary to the effect of the remaining adulterants where malt wort addition had a significant influence. Creep and recovery analysis were carried out, due to the tendency of honey solutions containing fructose, glucose and sucrose to crystallize as a result of the shearing. By computing a constant stress over time, creep parameters were determined, being expressed using the creep compliance function, and calculated through the Burgers model (Equation (19)) [51,56]. *G*_0_ values did not indicate any trend with the increasing of the adulteration agent, suggesting that adulteration did not affect the elastic strength. The same conclusion could be drawn from the values of *G*_1_ and *η*_1_.

However, the adulteration showed a significant influence on the Maxwell dashpot for honey samples adulterated with fructose, glucose and hydrolysed inulin syrup, as *η*_0_ increased with the increase of adulterant agent. The addiction of fructose to honeydew honey led to higher *J*(*t*) values, increasing the creep start point, whereas glucose, inverted sugar, malt wort and hydrolysed inulin syrup decreased the start point. For all samples, no recovery was observed after the stress removal during the recovery analysis, in agreement with the Newtonian behaviour, which was not altered by the addition of any of the adulterants. PCA, based on sugar composition (glucose, fructose, sucrose, maltose and melezitose) and the rheological parameters, allowed the identification of the authentic honeydew honey and the adulterated samples. From 5% of adulterant addition, rheological parameters confirmed their pertinence in honey authentication [51].

Amiry et al. [78] performed multivariate statistical analysis techniques, such as PCA and linear discriminant analysis (LDA), on physicochemical and rheological parameters of Iranian honey, to verify authenticity and detect and classify adulteration. Honey samples were mixed with date and invert sugar syrups, in concentrations between 7% and 30% (below the international standard threshold [1]), classified based on type and concentration of adulterant. The rheological evaluation comprised the measurement of viscosity, surface stickiness, stringiness, and texture. Surface stickiness was recorded as the maximum force needed to separate the sample from the used cylindrical probe, while stringiness was registered as the distance the probe departed from sample surface before the force dropped to a certain value (in this case, 2.5 g). After the dimensionality reduction of the original data by PCA, the potential of physicochemical and rheological properties to classify models and separate batches was evaluated by LDA. Despite the ability to detect the presence and concentration of adulterants, the rheological properties were less accurate than chemical and other physical properties [78]. In fact, 67.65% of Iranian honey samples were correctly identified using rheological properties, while more than 95% was achieved when using chemical and physical properties (for example, lactones, diastase activity, sucrose, free acidity, ash, HMF).

Belay et al. [75] discriminated Ethiopian honey through rheology, melisso-palynology and PCA, identifying the floral and geographical origin of 320 honey samples belonging to nine mono-floral sources obtained from beekeepers in diverse regions of the country [75]. With the highest bee density in Africa, Ethiopia has currently shown a growing interest in the commercialization and study of mono-floral honeys, also envisaging future developments in technological and therapeutic terms [75]. Chemical, flow, and sensorial properties of honey depend on its floral origin. However, even considering honey samples from the same geographical location and environmental conditions, literature studies point to significant differences in rheological properties [27]. A Newtonian time-independent behaviour was observed under the experimental conditions, in agreement with what is mostly observed in mono-floral honeys, despite deviations from Newton´s model as previously mentioned [2,23,31,32,47].

Belay et al. [75] also investigated the effect of temperature on the viscosity of Ethiopian mono-floral honey samples in the range 25–45 °C, applying the Arrhenius formalism to viscosity–temperature data. The contribution of moisture content and water activity (*a*_w_) to the viscosity of each mono-floral honey sample was also investigated, as honey viscosity tends to extensively decrease when moisture and *a*_w_ increase [18,75]. Considering the mono-floral dominance in the studied samples, which ranged from 59.8% (*Croton macrostachyus*) to 90.3% (*Schefflera abyssinica*), the results pointed to a time independent Newtonian behaviour with the highest and lowest viscosity (and also activation energy) recorded in *Eucalyptus globulus* and *Vernonia amygdalina* honeys, respectively (Table 1). Concerning *a*_w_ and moisture content, *Eucalyptus globulus* showed the lowest values, while the highest observed in *Schefflera abyssinica*. Based on the moisture content, *Vernonia amygdalina* honey should not have presented the lowest viscosity value of all honeys; yet viscosity also depends on honey floral origin (directly related to its composition). Even though the Ethiopian *Acacia* honey rheological behaviour seemed to agree with the Polish *Acacia* (black locust) honey [23], its viscosity values were higher in the operating temperature range (except at 25 °C) when compared with the Polish *Acacia*. The lower moisture content of the Ethiopian honey and the species variation of the two *Acacia* honeys could induce a difference in other honey components, which may explain the higher viscosity in the Ethiopian *Acacia* honey. In the case of *Eucalyptus* Ethiopian honey, a comparison with that of Algerian origin at 30 °C [83] showed a much higher viscosity value than the former, probably due to the influence of both moisture content and water activity. However, the contribution of the two physicochemical parameters to the viscosity of mono-floral honey samples proved to be distinct, and *a*_w_ showed a more pronounced effect; this fact could be related to the propensity of honey solid constituents to be attached to water molecules, decreasing the liquid phase of honey, and leading to an increase of viscosity with reduced *a*_w_ at different shear rates [75]. Interestingly, there has been a divergent rheological evaluation with respect to *Eucalyptus* honey. Although Ethiopian *Eucalyptus globulus* exhibited a Newtonian behaviour, other published works have reported a Newtonian [39] and a non-Newtonian [33,84] profile for the *Eucalyptus* spp., the latter probably ascribed to the presence of colloidal particles such as dextran [84].

Five kinds of Yichun honey from distinct floral origins (acacia, astragali, data, vitex, and buckwheat), all derived from the same geographical origin in China, and five kinds of acacia honeys from different Chinese geographical origins were classified by Wei et al. [67] applying multivariate analysis in the rheological analysis. The pattern recognition techniques comprised PCA, CA, PLS, and SVM. The viscosity values were obtained at three different temperatures between 20 and 40 °C, and all honeys confirmed Newtonian behaviour. Three regression models, PCR, PLSR and SVR were used for category forecast, and SVR appeared to be the best chemometric approach for all honey samples from the different floral and geographical origins. The correlations between the predicted value using SVR (an application of SVM for regression learning) and the experimental data obtained through a rheometer, showed the rheological potential for rapid assessment of the type of honey. However, honey samples from the different geographical origins were easier classified by the rheological technique, when compared to samples from floral origins.

The influence of honey botanical origins on rheological parameters was also investigated by Oroian et al. [64] using PCA, LDA and ANN to predict *G*′, *G*′′, η*, shear storage compliance *J*′ and shear loss compliance *J*′′. Fifty-one honey samples, botanically distinct (acacia, multifloral, sunflower, honeydew, and linden) and from Romania were characterised from both a rheological and physicochemical perspective, and the convenience of these properties for honey authentication was evaluated [64]. All honey samples displayed Newtonian behaviour, since the complex viscosity was not influenced by the frequency of originating a viscosity plateau. From the four chemical input parameters used for this ANN prediction (fructose, glucose, sucrose, and moisture content), moisture content presented the highest influence on the magnitude of the rheological parameters. Concerning the rheological properties, the best predictive power was found for *G*′′, η*, and *J*′′. Among the three statistical methods used, LDA proved to be the most suitable for honey botanical authentication.

Along with other physicochemical and sensory characteristics, Ribeiro et al. [47] determined the viscosity of Brazilian tiúba honey through steady-state measurements, aiming to evaluate the impact of different preservation techniques at 20 °C and 30 °C by 180 days. Tiúba is an indigenous stingless bee honey created by meliponi-culture that has a high moisture content (25%–30%) resulting in its deterioration by natural fermentation [47]. Preservation processes for maintaining sensory and nutritional quality and reported therapeutic effects generally cause modifications in the natural properties of honey [47]. Freezing induced a decrease in both moisture and viscosity of honey samples. Even considering that fluids’ viscosity is affected by moisture and temperature, the reduction of moisture by freezing did not lead to an increase of viscosity [47]. However, other factors such as concentration, form, and type of sugars, as well as other components present even in low concentrations (dextran, proteins, organic acids, vitamins, minerals, essential oils and pollen grains) also significantly affect viscosity values [47]. In contrast, Kedzierska-Matysek et al. [63] reported an increase in viscosity with freezing for raw rapeseed honey stored for 18 months, when compared to fresh control samples. Viscosity of Tiúba honey decreased with maturation when compared to the initial time samples, showing a greater decrease at the highest maturation temperature [47]. These results disagree with those described by Silva et al. [85] who observed that retention time had not significantly influenced the viscosity of *Apis mellifera* honey in a storage time of between 0 and 90 days.

The effect of temperature changes on the mechanical response of Brazilian honeys (mono-floral “assa-peixe”, “cipó-uva”, eucalyptus, orange blossom, and multifloral from southeast, south, northeast centre-west regions) was broadly examined resorting to rheological (shear and dynamic), total soluble solids (TSS) concentration analysis and DSC. The variation observed in the complex viscosity of honey samples clearly reflects the natural variation in their composition. Nevertheless, η* was always independent of frequency, indicating a liquid-like behaviour, and decreased with increasing temperature. Values of *η* also decrease with increasing temperature and its shear rate-independency denoted a Newtonian behaviour [39]. Among the forty honey samples, only two did not attend the Cox-Mertz rule (Equation (13)), due to deterioration of the material structure after the shearing, causing η* to be greater than *η*. At 10 and 15 °C, the best model applied to SR-SS data was the Power-law (Equation (2)), and honeys were classified as shear-thinning [52], but between 20 and 60 °C the best fit was achieved with the Newton´s equation [39,52].

Considering its relevant practical application, the combined effect of temperature (10–60 °C) and TSS concentration (79.52–84.48 °Brix) on *η* and η*, was examined by means of the Power-law and exponential models. The best statistical model (Equations (33) and (34)) was the same one obtained by Oroian et al. [38] when studying Spanish honeys in the 25–50 °C range, but with a higher *r*^2^ and different nonlinear regression parameters.

(33) $$\eta =7.74\cdot 10^{-32}\exp\left(0.447C+\frac{11099}{T}\right)$$

(34) $$\eta^{*}=6.55\cdot 10^{-32}\exp\left(0.427C+\frac{11637}{T}\right)$$

Comparing the activation energy of both prediction models, the value corresponding to the complex viscosity is higher, implying its higher sensitivity to temperature changes, and the superior susceptibility of the dynamic oscillatory measurements [39].

In another study [71], the authors employed ANN, namely MLP, to predict the rheological properties of the aforementioned Brazilian honeys [39,52], based on temperature and moisture content. Four independent ANN models were developed to predict *η* from steady shear measurements (model 1), *G*′, *G*′′ and η*, either from temperature scans during heating and cooling (models 2 and 3, respectively) or from frequency sweeps (model 4). Good predictive quality was obtained from all models, except for the rheological parameter *G*′ in models 3 and 4. The worst results observed for *G*′ expressed the high complexity and nonlinearity in its relationships with the input variables, in a similar behaviour already noticed by other authors [68]. Model 4 presented a superior predictive power when compared to a second-order polynomial regression model [71]. Overall, the obtained results pointed to the potential of ANN models to be applied for the processing of honey and honey-based products in engineering calculations and quality control domains [71].

Temperature effect on rheological properties of Portuguese honeys was studied for the first time by Afonso et al. [40], based on two uni-floral honeys, namely heather and rosemary, and a poly-flower honey. The Herschel-Bulkley (Equation (3)) and the Ostwald-De-Waele (Equation (2)) models were applied to the shear stress–shear rate data, and linear relationships were obtained indicating a Newtonian behaviour (except a weak shear thinning profile for rosemary honey at the highest temperature, which could possibly be imputed to experimental errors). Besides, no thixotropy was detected. For all analysed honeys, viscosity values decreased with increasing temperature, and the experimental data were fitted to an Arrhenius equation (Equation (20)) and to a three-parameter exponential empirical model. Rosemary honey displayed the lowest viscosity values at all temperatures (except at 95 °C), although it did not have the highest moisture content, and was the less temperature-sensitive honey with the lowest value for *E*_a_. Fitting the three-parameter exponential model to experimental data generated better correlations.

Rheological properties of the three most relevant Egyptian bee honeys (Citrus, Clover, and Marjoram) were measured in combination with a physicochemical characterization, to evaluate their quality [42]. When compared to the other honey samples, Marjoram honey presented the highest value of viscosity and shear stress. But for all the honey samples, viscosity decreased with increasing shear rate, manifesting its shear-thinning behaviour after deformation and rearrangement of particles, which reduces the flow resistance. In addition, the author observed a correspondence between a higher viscosity and a low water content in agreement with some literature reports [23,25,31,38].

Indian honeys had been referred to mostly as Newtonian systems [25,49,59,60,86], although there are some reports of non-Newtonian behaviour [31,59]. The results obtained by Naik et al. [60], who explored the rheological properties of three different raw and fresh Indian honey varieties of acacia, pine honeydew and multifloral honey samples, at 0–30 °C from Kashmir valley, indicated a Newtonian behaviour. Dynamic rheological measurements showed that viscous nature was much more pronounced than elastic in the three honey varieties, which is explainable by the differences in pollen spectra, sugar composition and moisture content. The values of viscous flow behaviour index, *n*, were equal to unity, implying the Newtonian response, irrespective of the geographical origin and temperature [60]. Gamma radiation (for preservation purposes) also did not alter the Newtonian character, as was observed by Saxena at al. [59] who characterized honey brands commonly available in the Indian market at 5–40 °C range.

The range of Arrhenius activation energy values obtained by Naik et al. [60] was similar to those already reported in the literature for honeys from other geographical locations. Acacia honey displayed the highest *E*_a_, i.e., the greater sensitivity to temperature effect, also showing the lowest *η*_0_ value. A similar viscosity–temperature dependency was found by Saxena et al. [59], as viscosity decreased with increasing temperature; however, at temperatures beyond 30 °C, the temperature effect was less pronounced which could be ascribed to natural variations in chemical composition. Other honey varieties from the Kashmir valley (saffron, apple, cherry and *Plectranthus rugosus)* were also investigated [25], in a pioneer study on the influence of temperature effects on honeys’ rheological properties. Experimental data of thirty-seven samples from the four honey varieties, obtained from dynamic frequency sweep tests, allowed the determination of *G*′ and *G*′′ and their dependence on angular frequency. As noticed before with honeys from the Kashmir valley [60], the viscous nature was much more pronounced than the elastic, and the temperature effect was only studied based on *G*′′, using the Power-law function. Again, all honey samples proved to be Newtonian fluids at the 0–30 °C range, despite their different floral origins. The temperature effect on viscosity seemed to be more significant at temperatures below 20 °C, as had been observed in the other honeys from Kashmir valley [60]. Saffron honey was found to be the variety with the lowest viscosity value (corresponding to the highest moisture content) and the highest *E*_a_, while *P. rugosus* showed the opposite trend [25].

In the presence of adulterants, Indian honeys have revealed a non-Newtonian behaviour as observed in adulterated honeys from other countries [49]. Kamboj and Mishra [49] noted that the viscosity of multifloral raw honey from Punjab increased linearly with the addition of jaggery (non-centrifugal sugar obtained from sugarcane juice, with the physical appearance of multifloral honey) at a particular temperature. Contrary to the pure honey, the flow behaviour of the adulterated samples was explained by the Bingham model, and a slight thixotropy was also noticed. With the adulteration, Arrhenius *E*_a_ increased due to the need for more energy by honey particles to overcome the viscous forces, and the highest *E*_a_ was achieved by the 30% adulterated honey. Oscillatory measurements were also performed to evaluate the effect of storage time on adulteration with 30% jaggery at 25 °C. The predominance of *G*′′ over *G*′ was only observed up to a particular frequency in both samples, after which *G*′ increased; in addition, *δ* was found to be above 45°, suggesting an elastic nature for the adulterated sample. Crossover point increased linearly with the percentage of adulterant increase. The results indicated that, in case of time delay, the elastic behaviour predominates over the viscous, leading to the total crystallization of honey at lower frequency [49].

Pridal et al. [45] analysed the rheological changes in heather honey, a favourite target of fraud, after its dilution with lime honey (a common honey). Results of the application of the Power-law (Equation (2)) and the Herschel-Bulkley (Equation (3)) models to SR–SS data showed that *n*, *K* and *τ*_0_ changed accordingly with the variation in the honey dilution. The variation of *n*-values (from both models) with percentage of mixing was non-linear, decreasing gradually with the amount of heather honey, but sharply from 40% (*w*/*w*) to 60% (*w*/*w*) heather honey. A similar tendency was observed for *K* (both models) and *τ*_0_. A clear shear-thinning behaviour was noticed from the 40% dilution.

Authors did not recommend the use of hysteresis area as an evaluation parameter for thixotropy, due to its great instability resulting from the sensitivity of the hysteresis loop to both internal and external factors [45]. Still regarding the dependence of the apparent viscosity versus time, the parameter *ϕ* (the ratio between *η* in the first second of the assay and *η* in the 300th second of the assay) followed the same trend observed for parameter *n*. After a gradual initial variation, authors observed a 13% change between samples with 40% and 60% heather honey. When applying the Weltman model (Equation (6)), a significative change of coefficient *B* values occurred again within the same concentration range. The frequency sweep test showed that the viscous module was predominant, and the largest and the smallest gap was observed for lime and heather honeys, respectively (Table 1). Parameter *C*, in the logarithmic dependence of η* on the angular frequency (Equation (35)) changed with the percentage of dilution.

(35) η*=C(lnω)+D

The highest negative value of *C* was noted for samples with 80% and 100% of heather honey, which suggests that they are better structured systems. Taking into account the variability of the data, the reliable parameters for the characterization of the diluted samples seemed to be the *n* values, from Power-law (Equation (2)) and Herschel-Bulkley (Equation (3)) models, and parameter *ϕ*. The non-linear dependence of the measured parameters on the degree of dilution, with a step change between 40% and 60% of dilution, could be used to identify adulterated heather honeys. However, it will be necessary to explore an eventual dependence of the step change on the choice of the diluent (concerning different types of honey or special sugar solutions, for example), and on the heather honeys from different geographical locations [45].

Although classical rheological measurements are responsive to temperature- and composition-dependent alterations in honey, information on changes (such as moisture absorption or sugar crystallization) are given by micro-rheology, which is elucidative about local heterogeneities and consequently about local rheology [20,87]. The ability to store and dissipate energy are examples of local mechanical properties which can be determined by way of small tracer particles embedded in the medium. The way these particles apply shear to the medium could be passively by Brownian motion (thermal fluctuations), or actively by an external force. In this context, Cohen and Weihs [20] investigated the temperature and composition dependence of four natural (NH) and reduced-calorie honey (RCH) samples, in terms of steady-state rheology and particle-tracking micro-rheology approaches for a better understanding of heterogeneities and local time-dependent changes in complex samples [20]. Selected floral Israeli honey varieties included citrus flower, wildflower, wildflower-based light (with polydextrose and sorbitol added) and field-flower-based light (with polydextrose added). In the video-based particle-tracking technique, fluorescent carboxyl-modified polystyrene sub-micron-sized particles were employed as probes and dispersed in each honey sample, and the particle dynamics were obtained by analysis of the time-dependent positions. A diffusive motion typical of a system in thermal equilibrium was observed in all honey varieties [20]. For complex fluids, it is necessary to know the relationship between Brownian motion and the viscoelastic properties of the environment, which can be accomplished by means of the Generalized Stokes-Einstein-Relation, GSER [87]. The Newtonian viscosity of the investigated honeys was calculated from the diffusion coefficient using the GSER, showing concordance with the viscosity calculated from the steady-state rheology, at all temperatures. RCH presented the highest water content, being less viscous than NH varieties at all temperatures (Table 1). The presence of polydextrose in RCH had little influence on their viscosity. The reduced viscosity of RCH could be imputed to the sugar replacement. Although sorbitol usually forms a network structure in several solvents, leading to a non-Newtonian behaviour, the analysed honeys maintained the Newtonian flow in a large range of SR, since the solubility of sorbitol in honey should be complete. The calculated Arrhenius *E*_a_ proved to be water content-dependent, presenting the lowest values for RCH. The variation in viscosity between honeys had mainly to do with the variation of water content and not with the additives. Results also confirmed the usefulness of combining rheology and micro-rheology in the case of homogenous Newtonian fluids. Thermal fluctuation particle-tracking micro-rheology (where the energy is at the scale of *k*_B_*T*, depending solely on the temperature of the matrix) can probe relatively high-viscosity samples (on the order of tens of Pa.s), providing localized measurements of honey rheology [20].

As the textural characteristics of viscous foods like honey can be evaluated with the back extrusion test, which is relevant in the quality control context, Maldonado et al. [41] compared both analyses using 26 samples of multifloral honeys from different geographical regions in Argentina (east central and north-east). Concerning the Arrhenius model (Equation (20)), all studied honeys required the same energy to flow (regardless of their distinct viscosity), and values of *E*_a_ were similar to those obtained for other honeys from different geographical origins [18,24,25,31,38,59]. Regarding the WFL equation (Equation (21)), the authors employed the “universal” constants, obtaining reasonable correlations in practically all samples, with *T*_g_ values not significantly different (in statistical terms) from the calorimetric. The values of *T*_g_ determined by Maldonado et al. [41] were higher than those obtained by Silva et al. [39], and Recondo et al. [18] using the “universal” constants, but very close in the case when the reduced coefficients were employed [18]. In other comparisons in the same conditions, *T*_g_ values proved to be higher than the ones obtained by Silva et al. [39], but in the same values range reported by Al-Malah [35] and Lazaridou et al. [24]. When authors allowed parameters *C*_1_ and *C*_2_ to vary and used calorimetric *T*_g_ as a reference temperature, a better correlation was achieved, obtaining values of *C*_1_ and *C*_2_ equivalent to those calculated by Lazaridou et al. [24]. The values of the constants *A* and *B* (Equation (23)) and *K* and *m* (Equation (24)), calculated in this work were quite different from those reported by Recondo et al. [18] and by Silva et al. [39], which despite having studied honeys from different botanical origins obtained similar values [18,39].

The differences found in the viscous nature of these Newtonian honeys were related to natural variations in honey composition. At equivalent numerical values of angular frequency and shear rate, *η* and η* were similar and the Cox-Mertz rule (Equation (13)) was obeyed, which is expected for Newtonian fluids without particle–particle interactions (reflected in the low values of *G*′). A higher value of hardness corresponded to a higher sugar concentration, in agreement with the rheological results. In fact, central Argentinian honeys, with higher sugar concentration, proved to be harder than those from the north region. On the other hand, values of *η*, *G*′′ and η* reflected the most pronounced viscous nature of honeys from the north region; in addition, F/G ratio was 1.54 for northern honeys, suggesting that these honeys remain liquid for longer times [41]. However, contrary to what Oroian et al. [74] reported, regarding the relationship between fructose content and textural parameters, fructose had no influence on the hardness of Argentinian honeys.

The temperature effect on the rheological properties of natural Polish honeys from different botanical origins was also analysed by Orczykowask et al. [88] but using phenomenological rheology methods. In phenomenological rheology, the mechanical state of the structure of a viscous fluid, principally as a function of temperature, is examined using fractional differential calculus. In this setting, authors determined the alterations in the rheological parameters of buckwheat honey, clover honey and coniferous honeydew honey, caused by temperature changes comparable to those that take place throughout storage and transport of honey. According to this methodology, which is based on the concept of a continuous medium, rheological parameters were determined from rheological measurements in the −20 to 70 °C range and from rheological dependencies. The former consisted of *G*′ and *G*′′ while the latter included *G*_e_ (equilibrium modulus or modulus of elasticity in the steady-state), *η* (Newtonian viscosity in the steady-state, calculated from *G*′′ and *ω*), $G_{N}^{0}$ (viscoelastic plateau modulus, determined from *G*′′ and *ω*, and identified with the power of crosslinking of the structure), *τ*_m_ (weight average relaxation time, related to the longest time gap), *τ*_0_ (average relaxation time, related to the shortest time gap), *J*_e_ (limit susceptibility in the equilibrium state), *ω*_0_ (crosslinking density of the structure), and *k* (mechanical vibration damping factor, which expresses the resistance of the medium’s internal structure to external vibrations).

The predominantly elastic or viscous nature of honeys depended on the temperature. For the three honeys, $G_{N}^{0}$ presented high values indicating that a medium was created with a strong structure typical of viscoelastic quasi-solid bodies, in the form of a pseudo-gel; the high values also implied the feasibility of decelerating the aging process of the medium [88]. In all honeys $G_{N}^{0}$ decreased with increasing temperature. A comparison analysis of $G_{N}^{0}$ showed that buckwheat honey (with the lowest moisture content and the lowest viscosity) was time resistant even at very low temperatures, while honeydew honey (with the lowest F/G ratio, the highest moisture content and the highest viscosity) showed better resistance to high temperatures and a decreasing trend in structural elasticity with increasing temperature. Structures of the Polish honeys also exhibited high values of *k* and *ω*_0_. The structure of clover honey (with the highest F/G ratio) proved to be more resistant to mechanical vibrations (like those occurring during transport) in the low temperature domain. On the other hand, honeydew honey’s structure was more resistant to mechanical vibrations in the temperature range 20 to 25 °C. The phenomenological rheology performed by Orczykowska et al. [88], that enabled the assessment of elasticity and the crosslinking ability of honey’s structure, could be applied to the design or the control of the processing steps, which is a pertinent issue when considering the preservation of physicochemical properties in the final product.

## 6. Conclusions

The importance of honey’s rheological properties is manifested by the high number of published research papers in recent years using honey from different countries and botanical sources. Data from the literature covered in this review shows that rheometry is a practical and promising technique for the assessment of honey types (based on botanical source and/or geographical location) and honey adulteration, which is relevant for honey authentication and certification. Similarly to other food products, identification of the rheological behaviour of honey has also provided valuable information for quality control and equipment selection regarding the handling, packaging and processing of honey.

The relations between honey’s physicochemical and rheological properties have been analysed through chemometric techniques, which proved to be powerful tools in revealing the complexity of these systems. Among these, PCA and ANN furnished interesting and reliable results. Despite the increasing number of studies on honey, many authors use a small sample matrix, sometimes unable to provide consistent relationships among the different evaluation parameters. Further studies should try to expand the number of samples, standardize experimental conditions for comparison purposes, and proceed to the optimization of the models, taking into account the accuracy of the rheometric measurements. This overview of rheological studies on honey may contribute to the development of honey-based products and can inspire future research in this field.

## Figures and Tables

**Table 1:** Rheological properties of honeys from different locations and botanical sources covered in this review.

Honey Variety and Geographical Origin	Viscometer/Rheometer Measuring Geometries	Rheological Methods and Variables Range	Measured Rheological Parameters	Main Outcomes	Ref.
Argentina: multifloral north (MFN), central (MFC)	Rheometer PP (gap 1.0 mm)	Preheating: 45 °C, 1 h SSF $\dot{\gamma }$: 0.1–400 s^−1^ *T*: 283.15–323.15 K DS—SAOS Frequency sweep: 0.4–600 rad s^−1^, *γ* 0.5% (LVR) *T*: 293.15 K	*η*, *σ* *G*′, *G*′′, η*	*σ* vs. $\dot{\gamma }$—Newtonian behaviour. *G*′′ >> *G*′*:* viscous nature (except at very high frequencies, *G*′′ = *G*′′). *η* ≅ η* (Cox-Merz rule verified). *η*, *G*′′, η* (MFN) > *η*, G′′, η* (MFC). *η* vs. *T* (Arrhenius): *E*_a_: 79.61 (MFN)—82.09 (MFC). *η* vs. *T* (WLF, *r*^2^ ≥ 0.81 MFN, ≥ 0.91 MFC); *C*_1_, *C*_2_—“universal” constants: *T*_g_: 224.59 K (MFN), 220.41 K (MFC) (matching *T*_g_ from DSC); *η*_g_: 1.32 × 10^11^ (MFN), 1.18 × 10^11^ (MFC). *η* vs. *T* (WLF with varying *C*_1_ and *C*_2_ constants and *T*_g_ from DSC, *r*^2^ ≥ 0.97 MFN; ≥ 0.96 MFC); *C*_1_: 13.75 (MFN), 14.63 (MFC); *C*_2_: 24.76 (MFN), 27.01 (MFC); *η*_g_: 1.95 × 10^18^ (MFN)—1.66 × 10^20^ (MFC). Agreement between rheology and back extrusion assays: hardness (MFC) > hardness (MFN); same consistency and adhesivity (MFN and MFC). Cluster analysis (rheological and textural parameters): weak classification of honeys.	[41]
Argentina: “algarrobo”	Rotational viscometer	SSF *T*: 278.15–343.15 K	*η*	Newtonian behaviour. *η* vs. *T* (Arrhenius, *r*^2^ = 0.994): *E*_a_: 82.8. *η* vs. *T* (WLF, *r*^2^ = 0.996); *C*_1_ (13.8), *C*_2_ (50); *η*_g_: 7.4 × 10^7^; *T*_g_: 227.95 K. *η* vs. *T* (VTF, *r*^2^ = 0.996); *B*: 1535. *η* vs. *T* (P-L, *r*^2^ = 0.998); *K*: 2.9 × 10^14^; *m*: 7.5. WFL equation with *C*_1_ and *C*_2_ calculated by reduced variables method: the most suitable for modelling viscosity-temperature dependence.	[18]
Australia: tulsi (TUL), manuka1 (MH1); USA: alfalfa (ALF); New Zealand: manuka2 (MH2)	Rheometer PP (*ϕ* 5 mm; gap 1 mm)	DS Samples equilibrated: 15 °C, 10 min; cooled (sub-zero region), 1 °C/min, 1 rads^−1^, γ = 0.01% Frequency sweeps: 0.1–100 rad s^−1^. *T*: 213.15–253.15 K	*G*′, *G*′′, *a_T_*, *T*_g_, *f*_g_, *α*_f_, *E*_a_	TTSP: production of a set of *a_T_*. log *a_T_* vs. *T* (Arrhenius-type fit), *E*_a_ = 108 (TUL), 86 (ALF), 81 (MH1), 99 (MH2). At upper temperature of the glass transition, log *a_T_* vs. *T* (WLF fit, modelling free volume): *C*_1_ = 10,70 (TUL), 10.85 (ALF), 11.43 (MH1), 11.13 (MH2); *C*_2_ = 50; *T*_g_ = 226.15 K (TUL), 228.15 K (ALF), 229.15 K (MH1), 227.15 K (MH2); (*T*_g_,_DSC_ = 226.15 K). *f*_g_ = 0.040 (TUL, ALF), 0.038 (MH1, MH2); *α*_f_ = 8.0 × 10^−4^ (TUL, ALF), 7.6 × 10^−4^ (MH1, MH2). Hbs in intermolecular association amongst monosaccharides generated a semi-crystalline system which allowed the prediction of mechanical *T*g, that define the passage liq-like to sol-like at sub-zero temperatures. WLF eq. allowed estimation of free volume parameters for honey vitrification.	[62]
Australia: blue top iron bark (BTIB), bloodwood (BDW), gum top (GT), heath (H), narrow leafed iron bark (NLIB), stringy bark NT (STB), tea tree (TT), yapunyah (YAP), yellow box (YB)	Rheometer with Couette geometry (*ϕ*_cup_ 34 mm; *ϕ*_bob_ 32 mm; *L*_bob_ 34 mm;	Preheating 55 °C, kept 30 °C SSF *T*: 275.15–313.15 K $\dot{\gamma }$~0.01–100 s^−1^	*η*	*η* = 1.0 (STB, 313.15 K)–410.7 (YAP, 275.15 K). *η* vs. *T* (Arrhenius, *r* ≥ 0.987): *E*_a_: 99.6 (TT)–106.0 (BDH); *η*_g_: 9.0 × 10^5^ (NLIB)—2.0 × 10^6^ (BTIB, BDW, H, STB). *η* vs. *T* (WLF, *r* ≥ 0.997); *C*_1_ (13.7–21.1), *C*_2_ (55.9–118.7); *η*_g_: 4.0 × 10^7^ (NLIB)–4.0 × 10^20^ (YAP). *η* vs. *T* (VTF, *r* ≥ 0.898); *B*: 4.5 (NLIB)–13.5 (YAP). *η* vs. *T* (P-L, *r* ≥ 0.951); *K*: 1.1 × 10^3^ (STB)–8.0 × 10^3^ (YAP); *m*: −2.3 (YAP)–2.2 (BDW). WLF: the most suitable model for viscosity-temperature dependence; constants *C*_1_ and *C*_2_ calculated from non-linear regression analysis, are valuable for adequate rheology modelling of honeys.	[8]
Brazil: *Hovenia dulcis* from *Apis mellifera* (Hd1) and *Tetragonisca angustula* (Hd2) bees	Rotational viscometer, cylindrical spindles, sample chambers	SSF $\dot{\gamma }$: 0–2.5 s^−1^ *T*: 303.15–333.15 K	*η, σ*	*η* (0.1 s^−1^): 0.08 (Hd2, 333.15 K)–45.50 (Hd1, 303.15 K). *η* vs. *T* (Arrhenius): *E*_a_: 52.65 (Hd2)–125.91 (Hd2). *σ* vs. $\dot{\gamma }$ (P-L), *r*^2^ ≥ 0.99; *K*: 5.22 (Hd2)–421.98 (Hd1); *n*: 0.88 (Hd1)–1.02 (Hd2). *σ* vs. $\dot{\gamma }$ (CA), *r*^2^ ≥ 0.98; *K*_C_: 2.36 (Hd2)–18.96 (Hd1); *σ*_C_ < 1.34. Hd1: Newtonian behaviour (303.15 K); non-Newtonian, shear thinning behaviour (313.15–333.15 K). Hd2: Newtonian behaviour (303.15 K); non-Newtonian, shear-thickening (313.15 K, 323.15 K), shear thinning behaviour (333.15 K).	[43]
Brazil: “assa-peixe” (AP), “cipó-uva” (CU), eucalyptus (EU), orange blossom (OB), multifloral—southeast (MF1), south (MF2), northeast (MF3), mid-west (MF4)	Rheometer PP (*ϕ* 1 mm; gap 35 mm)	Preheating 55 °C, kept 30 °C, 48 h SSF $\dot{\gamma }$: 0.1–100 s^−1^, 3 cycles *T*: 238.15–333.15 K DS—SAOS Stress sweeps, 1 Hz *f*: 0.1–10 Hz *T*: 283.15–333,15 K	*η* *G*′, *G*′′, η*	*η*: 147.3 (CU, 283.15 K)–0.35 (MF4, 333.15 K). η*: 151.33 (CU, 283.15 K)–0.42 (MF4, 333.15 K). *η* vs. η*: *α* ~ 1 (Cox-Merz rule verified), except: OB, MF1. *η* or η* vs. *T* (Arrhenius, *r*^2^ ≥ 0.994): *E*_a_ (*η*): 84.97 (CU)–92.53 (MF4); *E*_a_ (η*): 85.60 (EU)–100.40 (OB). *η* or η* vs. *T* (WLF, *r*^2^ ≥ 0.9999); with fixed *C*_1_ and *C*_2_ universal constants); *η*_g_ (*η*) 7.4 × 10^11^ (MF4)–1.09 × 10^12^ (CU); *T*_g_ (*η*) 210.47 K (MF4)–215.70 (CU); *η*_g_ (η*) 4.98 × 10^11^ (MF3)–1.63 × 10^12^ (AP); *T*_g_ (η*) 210.44 K (EU)–220.27 (OB); *η* or η* vs. *T* (VTF, *r*^2^ ≥ 0.9986); *B* (*η*): 1352.83 (MF4)–1465.71 (CU); *B* (η*): 1361.68 (EU)–1581.04 (OB). *η* or η* vs. *T* (P-L, *r*^2^ ≥ 0.9990); *K* (*η*): 1.83 × 10^15^ (MF4)—1.39 × 10^16^ (OB), *m* (*η*): 7.65 (MF4)–7.98 (OB); *K* (η*): 3.15 × 10^15^ (MF4)–6.34 × 10^17^ (MF1), *m* (*η*): 7.53 (EU)–8.63 (OB). η* independent of *ω*: liquid-like, Newtonian behaviour (293.15–333,15 K). Non-Newtonian, shear-thinning (283.15–288,15 K): WLF: best predictor model for OB, MF1, MF2. Increase in TSS concentration → increase in *E*_a_, *T*_g_ (WLF), *B* (VTF), *m* (P-L) coefficients. Selection of adequate T and TSS conditions, during processing and storage, are decisive for honey stability. ANN-MLP, input layers *T*, *ω*: *η* (model 1); *G*′, *G*′′, η* (model 2-heating; model 3-cooling). Input layers *T*, w, *ω*:); *G*′, *G*′′, η* (model 4). Potential application of the models (except for *G*´ in models 3 and 4), for the processing of honey and honey-based products.	[39,52,71]
Burkina Faso (north- and central-eastern)	Rheometer PP (*ϕ* 60 mm; gap 0.5 mm)	Preheating 55 °C, kept 30 °C DS Stress sweep, 1 Hz Frequency sweep: 0.62–62.83 rad s^−1^, 1 Pa (LVR) *T*: 278.15–313,15 K	*G*′, *G*′′, η*, *δ*	*G*′′ >> *G*′: viscous nature. η* vs. *ω*: constant function; *δ* ~90°: Newtonian behaviour. η* vs. *T*: *E*_a_ = 41.07–48.58. *G*′′ vs. *T*: *E*_a_ = 24.09–48.11. *E*_a_ (η*) ≅ *E*_a_ (*G*′′): Newtonian behaviour. Prediction of *G*′′ and η*: negative linear influence of fructose and temperature, positive linear influence of glucose.	[15]
Czech Rep.: blossom-honeydew (BHD), blossom honeydew lime (BHL), blossom honey nectar (BHN)	Rheometer CP, (*ϕ* 50 mm; angle 1°).	Preheating: 55 °C, 1 h; kept: 30 °C, 48 h SSF $\dot{\gamma }$: 0–100 s^−1^, *T*: 287.15–323.15 K	*η*, *σ*	*σ* vs. $\dot{\gamma }$ (Newton model): linear function. *η* (BHL) > *η* (BHD) > *η* (BHN) *η* vs. *T* (Arrhenius, *r*^2^ ≥ 0.9945); *E*_a_: 102.07 (BHN), 104.85 (BHD), 105.9 (BHL).	[46]
Egypt: citrus (CIT), clover (CLO), marjoram (MAR)	Viscometer CC	SSF $G_{N}^{0}$: 6.12–122 s^−1^ *T*: 298.15 K	*η, σ*	*η* vs. $G_{N}^{0}$: shear-thinning behaviour. *η*: 22.75 (MAR, w 18.10%, F/G 1.33); 12.50 (CLO, w 19.42%, F/G 1.27); 11.40 (CIT, w 19.74%, F/G 1.32).	[42]
Ethiopia: acacia (AC), *Becium grandiflorum* (BG), *Croton macrostachyus* (CM), *Eucalyptus globulus* (EUG), *Hypoestes* (H), *Leucas abyssinica* (LA), *Schefflera abyssinica* (SCA), *Syzygium guineense* (SG), *Vernonia amygdalina* (VA)	Rotational viscometer CC (*ϕ*_int_ 10.61 mm)	Preheating: 45 °C, 3 h + 50 °C, 30 min SSF $G_{N}^{0}$: 2.58–258.1 s^−1^ *T*: 298.15–318.15 K	*η*	*σ* vs. $G_{N}^{0}$ (Newton, *r*^2^ ≥ 0.96), *η*: 4.73 (CM, 318.15 K)–29.21 (EUG, 298.15 K) Newtonian behaviour. *η* vs. *T* (Arrhenius, *r*^2^ ≥ 0.96): *E*_a_: 9.859 (VA)–60.042 (EUG). *η* vs. *t*: constant function.	[75]
Germany: false acacia (FA), heather (H), sunflower (SF), lime (L), rape (R)	Rheometer CC	SSF $G_{N}^{0}$: 0.2–60 s^−1^ *T*: 283.15–323.15 K DS γ: 10^−3^ *T*: 273.15–348.15 K–273.15 K Heating/cooling rate: 1 K/min	*η, σ* *G*′, *G*′′, tan*δ*	*σ* vs. $G_{N}^{0}$ (Newton, FA), *η* = 0.841(323.15 K)–2.31 (313.15 K) *σ* vs. $G_{N}^{0}$ (P-L), *K*: 0.69 (SF, 323.15 K)–172.66 (L, 293.15 K); *n*: 0.800 (R, 303.15 K)–1.002 (R, 313.15 K). *σ* vs. $G_{N}^{0}$ (HB), *K*: 13.39 (H, 293.15 K)–620.06 (R, 283.15 K); *n*: 0.378 (R, 293.15 K)–1.001 (FA, 283.15 K); *σ*_y_*:* 0.15 (H, 293.15 K)–137.26 (R, 283.15 K). Newtonian (FA); Non-Newtonian (H, SF, L, R). *G*′′ >> *G*′ (FA, SF, L, R): viscous nature. *G*′ > *G*′′ (H): viscoelastic nature; heather honey: gel-like system after heating (>1.6% proteins in colloidal form). *T* = 338.79 K: *G*′ = 14.31; *G*′′ = 14.69; tan*δ* = 2.24. Crystallization of honeys is depended on botanic origin, temperature and storage time.	[30]
Greece: pine honeydew (PHD), fir honeydew (FHD), thymus (THY), orange blossom (OB), helianthus (HEL), cotton (COT)	Rotational viscometer CC, CC (*ϕ*_int_ 19.36 mm; *L*_int_ 58.08 mm*; ϕ*_ext_ 21 mm	Preheating: 45 °C, 3 h + 50 °C, 30 min SSF $G_{N}^{0}$: 5–100 s^−1^ *T*: 298.15–318.15 K	*η, σ*	*σ* vs. $G_{N}^{0}$—linear regression: Newtonian behaviour. *η*: 0.421 (COT, 318.15 K, w 21%)–26.52 (FHD, 303.15 K, w 15%). *η* vs. *T* (Arrhenius, *r*^2^ ≥ 0.9951): *E*_a_: 70.8 (COT, w 21%)–96.3 (FHD, w 15%).	[31]
Greece: pine honeydew (PHD), fir honeydew (FHD), multifloral (MF), orange blossom (OB)	Rheometer CC (*ϕ*_cup_ 28.92 mm; *ϕ*_bob_ 26.66 mm)	Preheating 50 °C, 1 h SSF *T*: 293.15–333.15 K $G_{N}^{0}$: 0.1–500 s^−1^ DS *γ*: 0.1% *ω*: 3–300 rad s^−1^ *T*: 293.15 K	*η, σ* *G*′, *G*′′, η*	*η* (293.15 K) = 9.9 (PHD)–200 (FHD). *σ* vs. $G_{N}^{0}$, constant viscosity: Newtonian behaviour. *G*′′ >> *G*′: viscous nature. *G*′: 0.15 (OB)–19.10 (FHD). *G*′′: 64 (OB)–1701 (FHD). η*: 7.7 (PHD)–167.0 (FHD). *η* and *G*′′ inversely related to the water content of honey. *η* vs. *T* (Arrhenius, *r*^2^ > 0.95), *E*_a_: 72.69 (PHD)–93.75 (FHD). *η* vs. *T* (WLF with fixed *C*_1_ and *C*_2_ universal constants, *r*^2^ = 0.95–0.99); *η*_g_: 3.3 × 10^11^ (PHD)—7.8 × 10^11^ (FHD)*; T*_g_: 209.88 K (OB)–230.53 (FHD). *η* vs. *T* (WLF with varying *C*_1_ and *C*_2_ constants and *T*_g_ from DSC, *r*^2^ = 0.95–0.99); *C*_1_: 17.20 (OB)–25.18 (PHD), *C*_2_: 13.95 (OB)–30.90 (PHD); *η*_g_: 7.1 × 10^12^ (OB)–5.2 × 10^21^ (PHD)*; T*_g_ (DSC): 225.85 K (PHD)–238.40 (FHD).	[24]
India: cotton (COT), coriander (COR), dalbergia (DAL), murraya (MUR)	Rheometer PP, (*ϕ* 50 mm; gap 0.5 mm)	Preheating: 50 °C, 1 h; kept: 30 °C DS Frequency sweep: 0.63–63 rad s^−1^, *γ* 0.5% (LVR) *T*: 278.15–303.15 K	*η*, *σ* *G*′, *G*′′	*η*: 3.89 (MUR)–185.13 (COR). *η* vs. *T* (Arrhenius, *r*^2^ ≥ 0.99); *E*_a_: 94.51 (COT)– 100.19 (COR). *G*′′: 227.4 (MUR, 303.15 k)–10.553 (COR, 278.15 K). *G*′′>>*G*′: viscous nature. *G* vs. *T* (Arrhenius; *r*^2^≥ 0.99); *E*_a_: 94.27 (COT)– 99.66 (COR). *G*′′ vs. *ω* (P-L), *r*^2^≥ 0.99; *K*′′: 3.70 (MUR, 303.15 K)–169.25 (COR, 278.15 K); *n*′′: 0.99–1. Newtonian behaviour.	[86]
India (Kashmir): saffron (SA), apple (AP), cherry (CH), *Plectranthus rugosus* (PR)	Rheometer PP, (*ϕ* 50 mm; gap 0.5 mm)	Preheating: 50 °C, 1 h, kept 30 °C DS Frequency sweep: 0.63–63 rad s^−1^, *γ* 3% (LVR) *T*: 273.15–303.15 K	*η*, *G*′, *G*′′	*η*: 0.35 (SA, 303.15 K)–21.97 (PR, 273.15 K). *G*′′ >> *G*′, *K*′′ >> *K*′*:* viscous nature. *G*′: 0.009 (AP, 303.15 K)–85.95 (CH, 273.15 K). *G*′′: 0.23 (SA, 303.15 K)–1382 (PR, 273.15 K). *G*′′ vs. *ω* (P-L), *r*^2^ ≥ 0.97; *K*′′: 0.37 (SA, 303.15 K)–22.02 (PR, 273.15 K); *n*′′: 0.96 (SA, 303.15 K)–1.00 (PR, 273.15 K). Newtonian behaviour. *η* vs. *T* (Arrhenius, *r*^2^ = 0.99): *E*_a_: 77.18 (PR)–85.59 (SA); *G*′′ vs. *T* (Arrhenius, *r*^2^ = 0.99): *E*_a_: 77.80 (PR)–86.88 (SA).	[25]
India: acacia (AC), pine honeydew (PHD), multifloral (MF)	Rheometer PP, (*ϕ* 50 mm; gap 0.5 mm)	Preheating: 50 °C, 1 h, kept 30 °C SSF $G_{N}^{0}$~0–1.8 s^−1^ *T*: 273.15–303.15 K DS Frequency sweep: 0.63–63 rad s^−1^, *γ* 3% (LVR) *T*: 273.15–303.15 K	*η, σ* *G*′, *G*′′	*σ* vs. $G_{N}^{0}$: Newtonian behaviour. *η*: 0.27 (AC, 303.15 K)–17.27 (MF, 273.15 K). *G*′′ >> *G*′, *K*′′ >> *K*′*:* viscous nature. *G*′: 0.01 (AC, 303.15 K)–15.3 (MF, 273.15 K). *G*′′: 0.19 (AC, 303.15 K)–1085.49 (MF, 273.15 K). *G*′′ vs. *ω* (P-L), *r*^2^: 0.99; *K*′′: 0.28 (AC, 303.15 K)–17.30 (MF, 273.15 K); *n*′′: 1. Newtonian behaviour. *η* vs. *T* (Arrhenius, *r*^2^ = 0.99): *E*_a_: 62.10 (PHD)–75.87 (AC).	[60]
India: multifloral honey, adulterated with jaggery (5–30%, w/w)	Rheometer PP, (*ϕ* 20 mm; gap 1 mm)	SSF $G_{N}^{0}$: 0–20 s^−1^, *T*: 298.15 K *σ*: 10 Pa, *T*: 278.15–303.15 K DS Frequency sweep: 0.1–40 Hz, σ 10 Pa, *γ* 0.409 (LVR) *T*: 298.15 K	*η*_app_, *σ* *G*′, *G*′′	*η*_ap_: 2.48 (5%)–4.83 (30%). *σ* vs. $G_{N}^{0}$ (Bingham model). Pure honey: Newtonian. Adulterated honey: non-Newtonian, Bingham plastic, anti-thixotropic. *η*_app_ vs. *T* (Arrhenius); *E*_a_: 35.48 (0%)–38.48 (30%). *G*′′>>*G*′: viscous nature. Adulteration only affected the viscous properties.	[49]
Iran: pure honey adulterated with data syrup (DS) and invert sugar syrup (IS)–7%, 15%, 30%	Rotating viscometer, and spindle Texture analyser; cylindrical probe (*ϕ* 25 mm; *ϕ* 6 mm; for adhesion-cohesion)	SSF *T*: 293.15 K $G_{N}^{0}$: 10 rpm *T*: 295.15 K	*η, F*_max_, adhesiveness, stringiness, Surface stickiness, *t*_Start-Stringiness_ *t*_stringiness_	Samples classification by PCA, LDA. LDA model based on rheological properties, detected and classified correctly 67.65% of honey samples adulterated with complex sugars.	[78]
Israel: citrus flower (CIT), wildflower (WF), wildflower-based light (WF-BL), field-flower-based light (FF-BL)	Rheometer CP, (*ϕ* 60 mm; angle 4°).	Preheating: 55 °C, 3 h; kept: 30 °C SSF $G_{N}^{0}$ > 0.001 s^−1^ *M* ≤ 40 *T*: 278.15–308.15 K VPT micro-rheology: Fluorescent, carboxyl-modified, polystyrene particles (*ϕ* 200 mm) embedded within honey samples	*η*	*η* vs. $G_{N}^{0}$–constant function: Newtonian behaviour *η* (natural honeys): 5.0 (WF, 308.15 K)–558.3 (CIT, 278.15 K). *η* (reduced calories honeys): 4.2 (FF-BL, 308.15 K)–193.8 (WF-BL, 278.15 K). *η* vs. *T* (Arrhenius, *r*^2^ ≥ 0.98); *E*_a_: 84.7 (FF-BL)–96.9 (CIT). >90% particles: diffusive motion, *α*_MSD_ = 1. *η*_microrheology_–calculated using the Stokes-Einstein relation. *η* ($G_{N}^{0}$) matched *η*_microrheology_–Newtonian behaviour in both length scales.	[20]
Jordan: common black horehound (CBH), globe thistle (GT), squill (Sq)	Rotational viscometer, CC (*ϕ* 15.2 mm; *L* 60 mm; gap width 5.8 mm	SSF $G_{N}^{0}$: 2.2–219.8 s^−1^ *T*: 293.15–323.15 K	*η, σ*	*σ* vs. $G_{N}^{0}$ (Newton, *r*^2^= 0.999), *η* = 0.84 (GT, 323.15 K)–52.12 (CBH, 293.15 K). *η* vs. *T* (Arrhenius, *r* ≥ 0.998)—*E*_a_: 95.64 (Sq), 97.56 (GT), 97.69 (CBH). *η* vs. *T* (WLF, *r* > 0.9995); *C*_1_, *C*_2_–“universal” constants: *T*_g_: 223.83 (Sq), 225 (GT), 228.44 (CBH); *η*_g_: 2.21 × 10^11^ (GT), 2.37 × 10^11^ (Sq), 2.62 × 10^11^ (CBH).	[35]
Mozambique (south-western): honeydew honey	Rheometer PP (*ϕ* 60 mm; gap 0.5 mm)	DS Stress sweeps, 1 Hz Frequency sweeps: 0.1–10 Hz, 1 Pa (LVR) *T*: 293.15–313,15 K	*G*′, *G*′′, η*	*G*′′ >> *G*′: viscous nature. G* vs. *ω*: constant function: Newtonian behaviour. ANN best models for the prediction of rheological parameters as a function of temperature, frequency, and chemical composition: MLP–for *G*′′ and η* (*r*^2^ > 0.950); PNN–for *G*′(*r*^2^ = 0.758). Sensitivity: *G*′′ and *G*′ to frequency and moisture; η* to moisture and temperature.	[69]
Norway: heather (H) Czech Rep.: lime (L) (H diluted with L, 10–80% *w*/*w*)	Rheometer, CP (*ϕ*_cone_ 50 mm; angle 1°, gap 0.103 mm)	SSF $G_{N}^{0}$: 1–100 s^−1^ *T*: 298.15 K DS Frequency sweep: 0.1–10 rad s^−1^, *γ* 1% (LVR) *T*: 298.15 K	*η*, *σ* *G*′, *G*′′, η*	*σ* vs. $G_{N}^{0}$ (P-L), *r*^2^ ≥ 0.999; *K*: 7.91 (L)–74.50 (H); *n*: 0.9924 (L)–0.6745 (H). *σ* vs. $G_{N}^{0}$ (HB), *r*^2^ ≥ 0.999; *K*: 8.0 (L)–61.0 (H); *n*: 0.989 (L)–0.713 (H) *σ*_y_: (-)1.15 (L)–44.94 (H). *σ* vs. *t* (Weltman), *r*^2^: 0.55–0.84; *B*: [-]4.5 (L)–28.0 (H). *ϕ* ($G_{N}^{0}$ 50 s^−1^): 1.0534 (L)–0.7054 (H). *C* (Equation (35)): [-]1.93 (L)–[-]15.7 (H). Non-linear dependence of rheological parameters (*K*, *n* (P-L), *K*, *n* (HB), *σ*_y_, *B*, *ϕ*, *C*) on the degree of dilution with a step change between 40% and 60% (*w*/*w*): possible use in the identification of adulterated heather honeys.	[45]
Poland: buckwheat (BW), clover (CLO), honeydew (HD)	Rheometer PP, (*ϕ* 50 mm; gap 0.5 mm)	Preheating: 50 °C, 3 h; kept: 30 °C, 24–48 h DS Frequency sweep: 0.1–100 rad s^−1^, *γ* 1% (LVR) *T*: 253.15–343.15 K	*η*, *G*′, *G*′′	*η* (343.15 K): 13.5 (BW, wt 10.21)–324 (HD, wt 16.72). BW: *G*′ > *G*′′ (303.15 K, 313.15 K); *G*′′ > *G*′ (263.15 K, 343.15 K); *G*′ = *G*′′ (253.15 K, 258.15 K, 283.15 K, 293.15 K, 323.15 K, 333.15 K). CLO: *G*′′ > *G*′ (263.15 K, 293.15 K, 333.15 K, 343.15 K); *G*′ > *G*′′ (313.15 K, 323.15 K); *G*′ = *G*′′ (253.15 K, 258.15 K, 283.15 K, 303.15 K). HD: *G*′′ > *G*′ (268.15–293.15 K, 343.15 K); *G*′ > *G*′′ (323.15 K, 333.15 K); *G*′ = *G*′′ (253.15–263.15 K, 303.15–313.15 K). Rheological parameters of the phenomenological method: *G*_e_, *J*_e_, $G_{N}^{0}$, *τ*_m_,*τ*_0_, *ω*_0_, *k*. High values of $G_{N}^{0}$ (~10^1^−10^7^), *ω*_0_ (~10^−2^−10^2^), *k* (~10^0^−10^4^): honeys with structure of quasi-solid bodies, tending to form a pseudo-gel (high total elasticity, high cross-linking density and capacity); structure able to damp mechanical vibrations; structure sensitive to changes caused by temperature; structure able to slow down the physical aging of honey systems over time. Usefulness in the design and prediction of processing steps.	[88]
Poland: rape (R), multifloral (MF), buckwheat (BW). *a*) liquefied (55 ºC, 24 h + cooling, RT); *b*) crystallised	Rheometer CC (*ϕ*_int_ 26.652 mm; *ϕ*_ext_ 28.905 mm; gap 1.127 mm	*T*: 293.15 K *(a*) liquefied SSF—*σ*: 0–500 Pa DS—*ω*: 0–250 s^−1^ *(b*) crystallised SSF $G_{N}^{0}$: 0–450 s^−1^ *t*: 0–180 s (up- and downward) DS–(the same as in the liquefied samples)	*η* G*, *δ*, η* η, σ G*, *δ*, η*	*(a*) liquefied *σ* vs. $G_{N}^{0}$ (Newton, *r*^2^ = 0.999), *η* = 6.66 (R), 5.02 (MF), 3.18 (BW). G* vs. *ω* (*r*^2^ > 0.995), G* = 6.889*ω* (R), 4.794*ω* (MF), 2.894*ω* (BW). *(b*) crystallised Hysteresis area: large (R, MF), insignificant (BW). *σ* vs. $G_{N}^{0}$ (P-L, *r*^2^ ≥ 0.98): *K* = 36.696 (R), 15.945 (MF), 6.2218 (BW); *n* = 0.623 (R), 0.706 (MF), 0.854 (BW). G*(MF) > G*(R) > G*(BW). η* vs. $G_{N}^{0}$ (P-L, *r*^2^ ≥ 0.900): *K* = 374.86 (MF), 252.06 (R), 193.81 (BW). SSF results differ from DS measurements. Structural and rheological properties of the final product may be modelled by controlling the crystallization process.	[11]
Poland: rape-seed (stored for 18 months)	Universal Testing Machine with back extrusion cell (*ϕ* 50 mm; *L* 60 mm)	4 cycles: 50–400 mm/min *(a*) CON; *(b*) RT, *(c*) FRO	*η*	*η* = 33.6 (CON), 78.0 (RT), 280.5 (FRO) Storage temperatures influenced honey viscosity. The higher viscosity of FRO honey is probably a result of a crystallized structure formed by fine crystals.	[63]
Poland: heather	Rheometer PP (*ϕ* 35 mm; gap 0.5 mm)	Preheating 40 °C SSF $\dot{\gamma }$:1–100 s^−1^ (up- and downward), *t* = 180 s *T*: 283.15–313.15 K DS *T*: 283.15–313,15 K *ω*: 1–100 rad s^−1^ *γ*: 0.03	*η*, *σ* *G*′, *G*′′, η*	*σ* vs. $\dot{\gamma }$ (HB), *r*^2^ ≥ 0.999; *K*: 2.0–108.6; *n*: 0.66–0.90; *σ*_y_*:* 2.3–142.2 *K* vs. *T* (Arrhenius)—*E*_a_: 47.7–71.7. *σ* vs. *t* (Weltman), *r*^2^ ≥ 0.96; *B* [[–]: 10.7–56.7. *G*′′>>*G*′: viscous nature. *G*′′ vs. *ω* (P-L), *r*^2^ ≥ 0.9990; *K*′′: 2.6–163.4; *n*′′: 0.78–0.94. *η* vs. η* (P-L); *K*: 0.017–0.264; *β*: 1.39–2.11. Significant dependence of η* on *ω*: viscoelastic nature. Non-Newtonian, shear-thinning, tendency to yield stress, thixotropic.	[32]
Poland: acacia (AC), buckwheat (BW), linden (LI), multifloral (MF), rape (R), honeydew (HD), nectar-honeydew (NHD)	Rheometer (*ϕ*_cup_ 15.8 mm; *ϕ*_bob_ 14.00 mm)	Preheating 50 °C, 3 h SSF *T*: 283.15–313.15 K $\dot{\gamma }$: 1–100 s^−1^ Time effect: *T*: 293.15 K, $\dot{\gamma }$: 50 s^−1^	*η*	*η* = 1.8 (BW, MF, R–313.15 K)–252.6 (NHD, 283.15 K). *η* vs. *T* (Arrhenius, *r*^2^ ≥ 0.997): *E*_a_: 92.34 (BW)–105.25 (NHD). *η* vs. *T* (WLF, *r*^2^ > 0.999); universal constants *C*_1_, *C*_2_; *η*_g_: 1.88 × 10^11^ (R)–2.86 × 10^11^ (BW) *T*_g_: 220.34 K (BW)–228.39 (NHD).	[23]
Portugal: heather (H), rosemary (ROM) multifloral (MF)	Rotational viscometer, CC, spindles (*ϕ* 1.18 cm; *ϕ* 0.94 cm)	SSF $\dot{\gamma }$ ~ 0.2–60 s^−1^ Up- and downward *T*: 303.15–368.15 K	*η*, *σ*	*σ* vs. $\dot{\gamma }$ (HB), *r*^2^ ≥ 0.976; *K*: 0.05 (H, 368.15 K)–136 (MF, 303.15 K); *n*: 0.852 (ROM,368.15 K)–1.68 (H, 368.15 K); *σ*_y_ *<* 8.5 (insignificant effect of microparticles (crystals) in honey. *σ* vs. $\dot{\gamma }$ (P-L), *r*^2^ ≥ 0.956; *K*: 1.23 (ROM, 343.15 K)–139.8 (MF, 303.15 K); *n*: 0.849 (ROM,368.15 K)–1.105 (MF, 303.15 K). *η* = 74 (MF, 368.15 K)–13,678 (MF, 303.15 K). η* vs. *T* (Arrhenius, *r* ≥ 0.946), *E*_a_: 57.7 (ROM)–74.5 (MF). η* vs. *T* ($=K_{0}\cdot e^{A/T-B}$ fit, *r*^2^ ≥ 0.9999). Newtonian behaviour, except ROM 368.15 K (slightly shear-thinning).	[40]
Romania: honeydew (HD), adulterated with fructose (F), glucose (G), hydrolysed inulin (I), malt wort (M), inverted sugar (IS), (5–50%, w/w).	Rheometer PP, (*ϕ* 60 mm; gap 1 mm)	SSF $\dot{\gamma }$: 0–100 s^−1^ (up- and downward) *T*: 293.15 K DS Stress sweeps: 1 Hz, *σ* 1 Pa (LVR) *ω*: 0.62–62.83 rad s^−1^ *T*: 293.15 K	*η*, *σ* *G*′, *G*′′	*σ* vs. $\dot{\gamma }$ (Newton, *r*^2^ ≥ 0.999), Newtonian behaviour. *η* (HD): 16.64; *η* (5–50%)—HD+F: 16.02–11.58; HD+G: 17.02–21.94; HD+I: 16.24–21.22; HD+M: 16.64–16.71; HD+IS: 16.63–16.56. *η* vs. $\dot{\gamma }$, thixotropic area: increased by M, G, S, IS (highest, HD+M); decreased by F. *G*′′ >> *G*′: viscous nature. *G*′′ vs. *ω* (P-L), *r*^2^ ≥ 0.999; *K*′′; *n*′′: 16.78; 0.991 (HD); 16.27–11.76; 0.990–0.988 (HD+F); 17.25–22.15; 0.991–0.993 (HD+G); 16.62–21.40; 0.994–0.995 (HD+I); 17.47–22.92; 0.986–0.951 (HD+M); 16.85–16.70; 0.991–0.996 (HD+IS). Creep phase: 0–180 s. *J* vs. *t* (Burgers model, *r*^2^ ≥ 0.983): significative influence of F, G, I on *η*_0_ (~10^3^–10^7^). Creep start point: F increases, IS decreases. Recovery phase: 180–360 s; *J* vs. *t*: Newtonian behaviour; no influence of the adulterants. Honey authentication: PCA (rheological parameters + sugar composition): 100% explanation of total variance.	[51]
Romania: linden (LI), black locust (BL), rape (R), sunflower (SF), honeydew (HD), multifloral (MF)	Rheometer, PP (*ϕ* 40 mm; angle 2°, gap 1 mm)	SSF $\dot{\gamma }$: 0.1–500 s^−1^ (up- and downward) *T*: 283.15–313.15 K DS Frequency sweep: 3–300 rad s^−1^, *γ*: 3% (LVR) *T*: 293.15 K	*η*, *σ* *G*′, *G*′′, *δ*	*σ* vs. $\dot{\gamma }$—Newtonian behaviour: LI, BL, SF, MF; non-Newtonian with thixotropy: R, HD. *η* (293.15 K): 17.2 (HD)–2.7 (LI). *G*′′ >> *G*′: viscous nature. *G*′: 13.8 (LI)–315.6 (SF). *G*′′: 610 (LI)–2229 (SF). tan*δ*: 55–161. LDA to predict viscosity based on carbohydrate composition, *p*-value < 0.05: glucose and fructose; correct classification of 78.8% samples.	[21]
Spain: eucalyptus (EU), honeydew (HD), orange (OB), multifloral (MF), rosemary (ROM), summer savoury (SS)	Rheometer CC. Rheometer PP, (*ϕ* 60 mm; gap 0.5 mm)	Preheating: 55 °C; kept: 30 °C SSF $\dot{\gamma }$: 0–100 s^−1^ *T*: 298.15–323.15 K DS Stress sweeps: 1 Hz Frequency sweep: 0.628–62.8 rad s^−1^, *σ* 1 Pa (LVR) *T*: 298.15–323.15 K	*η*, *σ* η*	*σ* vs. $\dot{\gamma }$ (Newton, *r*^2^ ≥ 0.99), Newtonian behaviour. *η*: 0.462 (ROM, 323.15 K)–13.970 (HD, 298.15 K). *η* vs. $\dot{\gamma }$ and η* vs. $\dot{\gamma }$: constant functions–Newtonian behaviour. *η* vs. η*, Cox-Merz rule verified (*α*~1): all honeys at 313.15–323.15 K; except 298.15–303.15 K (*α* > 1). Prediction of *η* and η* from each other, through a modified Cox-Merz rule. *η* vs. *T* (Arrhenius, *r*^2^ ≥ 0.998); *E*_a_: 84.07 (ROM)–91.35 (HD). *η* vs. *T* (VTF), *r*^2^ = 0.999); *B*: 1595 (OB)–1954 (HD). *η* vs. °Brix and *T*: P-L and exponential models (*r*^2^: 0.733, 0.822); *E*_a_: 73.317, 82.773.	[38]
Spain: honeydew (HD), orange (OB), multifloral (MF), rosemary (ROM)	Rheometer PP, (*ϕ* 60 mm; gap 0.5 mm)	Preheating: 55 °C; kept: 30 °C DS Stress sweeps: 1 Hz Frequency sweep: 0.1–10 Hz, *σ* 1 Pa (LVR) *T*: 278.15–313.15 K	*G*′, *G*′′, η*	*G*′′>>*G*′: viscous nature. η* vs. *ω*: constant function–Newtonian behaviour. *G*′′ vs. *ω* (P-L), *r*^2^ ≥ 0.99; *K*′′: 1.13 (ROM, 313.15 K)–215.74 (HD, 273.15 K); *n*′′: 0.99–1.05. Application of TTSP to viscoelastic properties: obtention of a viscoelastic model (4th grade polynomial equation, *r*^2^ > 0.99), suitable for all honeys.	[54]
Spain: rosemary (RO) *(a*) liquefaction by heating (HT) *(b*) liquefaction by ultrasound (US)+HT	Viscometer, disc-type	HT: 313.15–333.15 K, 60 min US: 40 Hz, 313.15–333.15 K, 60 min. $\dot{\gamma }$: 2.5–20 rpm *t*: 20–60 min	*η*	*σ* vs. $\dot{\gamma }$, constant viscosity: Newtonian behaviour. *η* (HT) = 333 (333.15 K, 60 min)–3240 (313.15 K, 10 min). *η* (US) = 206 (333.15 K, 60 min)–3080 (313.15 K, 10 min). *η* vs. *T* (Arrhenius)—*E*_a_ (HT): 64; (US): 59. HT/US, 60 min—*η*: 1494/833 (313.5 K); 726/290 (323.5 K); 333/206 (333.5 K). At a same temperature and after a certain period of time, *η* of US samples are lower; honey can be liquefied by US, without the need to increase temperature up to 323.15 K or higher temperatures.	[13]
Spain: “Miel de Galicia”	Rotational viscometer CC	Preheating: 55 °C; kept 30 °C. SSF $\dot{\gamma }$: 0.3–2 s^−1^ (up- and downward) *T*: 298.15 K *T*: 280.15–328.15 K $\dot{\gamma }$: 1.4 s^−1^	*η*, *σ*	*σ* vs. $\dot{\gamma }$ (P-L): *K* = 7.887 × 10^−3^– 14.279 × 10^−3^; *n* = 0.933–0.969. Shear-thinning behaviour (at low $\dot{\gamma }$ values). *η* vs. *T* (Arrhenius; the best regression): *E*_a_: 83.880–96.631. *η* vs. *T* (WLF); *C*_1_ [-] 54.4–32.2), *C*_2_ 73.1–194.0; *η*_g_: 1.1 × 10^6^—1.2 × 10^9^ *η* vs. *T* (VTF), *r*^2^ = 0.996); *B*: 875.85–992.09. *η* vs. *T* (P-L); *K*: 4.96 × 10^16^–1.83 × 10^18^ -; *m* [-]: 9.25–8.57. Temperature effect more relevant in the low range of temperature.	[2,58]
Tunisia: eucalyptus (EU), orange (OB), rosemary (ROM), thyme (TH), mint (MI), horehound (HH)	Rheometer CP, (*ϕ* 35 mm; gap 0.14 mm)	SSF $\dot{\gamma }$: 0.01–500 s^−1^ *T*: 293.15 K DS Frequency sweep: 0.1–100 rad s^−1^, *σ* 0.001 Pa *T*: 293.15–323.15 K	*η*, *σ* *G*′, *G*′′, η*	*σ* vs. $\dot{\gamma }$ (HB, *r*^2^ ≥ 0.99), *K*: 8.47 (HH)–36.23 (TH); *n*: 0.68 (TH)–0.86 (HH); *σ*_y_*:* 3.72 (HH)–41.18 (TH). Non-Newtonian, shear-thinning behaviour. *η*_app_ vs. *T* ($\dot{\gamma }$: 10 s^−1^, Arrhenius, *r*^2^ ≥ 0.97); *E*_a_: 21.23 (HH)–34.91 (TH). *σ* vs. *t* (Weltman, *r*^2^ ≥ 0.97); *B*: 8.64 (HH)–21.10 (TH). *G*′′>*G*′: viscous nature. *G*′′ vs. *ω* (P-L), *r*^2^ ≥ 0.96; *K*′′: 0.65 (HH, 323.15 K)–143.10 (TH, 293.15 K); *n*′′: 0.79 (TH, 323.15 K)–0.91 (TH, 293.15 K); non-Newtonian behaviour.	[33]
Turkey: creamed honey	Rheometer PP, (*ϕ* 50 mm; gap 0.5 mm)	SSF $\dot{\gamma }$: 1–70 s^−1^, *T*: 283.15 K $\dot{\gamma }$: 1–100 s^−1^, *T*: 298.15–313.15 K; (up- and downward) DS Frequency sweep: 0.1–10 Hz, *γ* 0.5% (LVR) *T*: 283.15–313.15 K Temperature sweeps: *γ* 0.5% (LVR), 1 Hz, *T*: 278.15–323.15 K Thermal Loop 11 thermal cycles: 278.15–323.15 K, 10 rad s^−1^, *γ* 0.5%	*η*_app_, *σ* *G*′, *G*′′, η*	*σ* vs. $\dot{\gamma }$ (P-L), *r*^2^ ≥ 0.9993; *K*: 269.7 (283.15 K)–10 (313.15 K); *n*: 0.7641 (283.15 K)–0.8124 (313.15 K). *Hysteresis Area*: 51,713 (283.15 K)–1129 (313.15 K). *η*_app_,*_50 s−1_* vs. *T* (Arrhenius, *r*^2^ ≥ 0.9188); *E*_a_: 36.62. *G*′′>>*G*′: viscous nature. *G*′′ vs. *T* (Arrhenius, *r*^2^ ≥ 0.8565); *E*_a_: 41.71. *G*′′ vs. *t* (Weltman), *r*^2^≥ 0.9541; [-] *B*: 298.7 (283.15 K)–17.1 (313.15 K *G*′′ vs. *ω* (P-L), *r*^2^≥ 0.9926; *K*′′: 273.4 (283.15 K)–4.0 (313.15 K); *n*′′: 0.881 (283.15 K)–1.033 (313.15 K). Non-Newtonian shear-thinning thixotropic behaviour. Δ_min_ (*G*′′): 1.00 (cycle 1)–0.566 (cycle 11). Creamed honey with low thermal stability: great structural change by thermal stress.	[29]
Turkey: natural honey adulterated with saccharose (HAS) and fructose (HAF) syrups (0–50%, *w*/*w*)	Rheometer PP (*ϕ* 50 mm; gap 0.5 mm)	SSF $\dot{\gamma }$: 0.1–100 s^−1^, *T*: 298.15 K DS *T*: 298.15 K Amplitude sweep test, 1 Hz, γ: 0.1–100% Frequency sweep test, 1% (LVR), 0.1–10 Hz Temperature sweep test, 278.15–323.15 K, 1 Hz, 50 s^−1^ Creep phase: 0–150 s; recovery phase: 150–300 s	*η*, *σ* *G*′, *G*′′, G*	*σ* vs. $\dot{\gamma }$ (Newton), *r*^2^ ≥ 0.996 (HAS), *r*^2^ ≥ 0.997 (HAF): Newtonian behaviour. *η* = HAS: 6.531 (0%)—2.019 (50%); HAF: 6.531 (0%)–1.085 (50%). *G*′′ >> *G*′: viscous nature. *G*′′ vs. *ω* (HAS, *r*^2^ = 0.999; HAF, *r*^2^ ≥ 0.998); Newtonian behaviour *K*′′ = HAS: 6.367 (0%)–2.234 (50%); HAF: 6.367 (0%)–1.111 (50%); good indicator to detect honey adulteration at levels 10–50%, within a 278.15–323.15 K range. K*: same results as *K*′′; natural honey with the highest total resistance to deformation. *J* vs. *t* (Burgers model: *r*^2^ = 0.999 (HAS, HAF); *η*_0_ = HAS: 2.0–7.0; HAF: 1.1–7.0. *G*_0_, *G*_1_, *η*_1_: no consistent trend with increasing adulterant level; cannot be used to detect adulteration. *η*, *G*′′, η*, *η*_0_: potential to be good indicators of adulteration with saccharose and fructose, at specified ratios. 93.879% of the total variance in data set was described by four Principal Components, regarding physicochemical and rheological properties of natural and adulterated honeys.	[56]

ANN, artificial neural networks; *a_T_*, vertical shift factor; *b_T_*, horizontal shift factor; *B*, constant of the VTF model for temperature dependence (K); *C*, parameter in Equation (35)*; C*, concentration; *C*_1_, coefficient in the WLF model; *C*_2_, coefficient in the WLF model (K); CA, Casson model; CC, concentric cylinders; CP, cone-and-plate; CON, control (fresh honey); *D*, diffusion coefficient; DSR, dynamic shear rheology; *E*_a_, Arrhenius activation energy for flow (kJ mol^−1^); F/G, fructose/glucose ratio; *F*_max_, maximum force required to separate the probe from the sample; *f*, frequency (Hz); *f*_g_, fractional free volume at *T*_g_ (WLF equation); FRO, in freezer (−20 °C); *G*_0_, instantaneous elastic modulus of the Maxwell unit, in the Burgers model; *G*_1_, shear modulus of the Kelvin-Voigt unit, in the Burgers model; *G*′, storage modulus (Pa); *G*′′, loss modulus (Pa); G* complex modulus (Pa); *G*_e_, equilibrium modulus (modulus of elasticity in the steady state, Pa); $G_{N}^{0}$, the viscoelastic plateau modulus (power of crosslinking of the structure, Pa); HB, Herschel-Bulkley model; HBs, Hydrogen bonds; *J* (t), creep compliance (shear); *J*_e_, limit susceptibility in the equilibrium state (Pa^−1^); *K*, consistency in Power-law model for viscosity (Pa s^n^); *K*, constant of the P-L model for temperature dependence (Pa.s); *K*′, elastic intercept in power law model (Pa s^n^); *K*′′, viscous intercept in power law model (Pa s^n^); *K*_C_, Casson plastic viscosity; *k*, mechanical vibration damping factor; LAOS, large amplitude oscillatory shear; LDA, linear discriminant analysis; LVR, linear viscoelastic region; *m*, constant of the P-L model for temperature dependence; *M*, torque (N m); MLP, multilayer perceptron; MSD, mean-square displacement; *n*, flow behaviour index in power law model for viscosity; *n*′, elastic slope in power law model; *n*′′, viscous slope in power law model; NH, natural honey; PCA, principal component analysis; P-L, power-law model; PNN, probabilistic neural network; PP, parallel-plates; PS, polystyrene; *R*, gas constant (kJ mol^−1^ K^−1^); RCH, reduced calorie honey; rpm, revolutions per minute; RT, room temperature (20–26 °C); SAOS, small amplitude oscillatory shear; SSF, steady shear flow; tan*δ*, loss tangent; *t*, shearing time (s); *t*_Start-Stringiness_, time from withdrawal until the string tore; *t*_stringiness_, time corresponding to the distance the probe moved away from the sample surface before the force dropped to 2.5 g; *T*, absolute temperature (K); TTS, total soluble solids (°Brix); TTSP, time–temperature superposition principle; VTF, Vogel–Tamman–Fulcher model; VPT, video particle tracking; w, weight; WLF, Williams-Landel-Ferry model; wt, water content. *α*, shift factor (Cox-Merz rule); *α_f_*, thermal expansion coefficient (deg^−1^) above *T*_g_ (WLF equation); *α*_MSD_, MSD scaling exponent; *β*, constant of the Power-law function; *δ*, phase angle; Δ, relative structural index; *η* ($\dot{\gamma }$), steady shear viscosity; *η*_0_, zero-shear viscosity; *η*_0_ is the viscosity of the liquid filling the dashpot of the Maxwell element in the Burgers model (Pa s); *η*_1_, viscosity of the liquid filling the dashpot of the Kelvin-Voigt element, in Burgers model (Pa s), *η*_app_, apparent viscosity; *η*_g_, viscosity at *T*_g_ (Pa.s); η*, complex viscosity; *ϕ*, diameter; *ϕ*, proportion of the *η*_app_ in the 1st second of the assay (Pa.s) and the *η*_app_ in the 300th second of the assay (Pa.s); *γ*, shear strain; $\dot{\gamma }$, shear rate (s^−1^); *σ*, shear stress (Pa); *σ*_C_, Casson yield stress (Pa); *σ*_y_, yield stress (Pa); *τ*_0_, number average relaxation time (s);*τ*_m_, weight average relaxation time (s); *ω*, angular frequency (rad s^−1^); *ω*_0_, cross-linking density of the structure (rad s^−1^).

## Data Availability

Not applicable.
